# The nascent polypeptide-associated complex subunit Egd1 is required for efficient selective mitochondrial degradation in budding yeast

**DOI:** 10.1038/s41598-023-50245-7

**Published:** 2024-01-04

**Authors:** Yuan Tian, Koji Okamoto

**Affiliations:** Laboratory of Mitochondrial Dynamics, Graduate School of Frontier Biosciences, Osaka University, Suita, Osaka 565-0871 Japan

**Keywords:** Mitophagy, Mitochondria

## Abstract

Selective degradation of dysfunctional or excess mitochondria is a fundamental process crucial for cell homeostasis in almost all eukaryotes. This process relies on autophagy, an intracellular self-eating system conserved from yeast to humans and is thus called mitophagy. Detailed mechanisms of mitophagy remain to be fully understood. Here we show that mitochondrial degradation in budding yeast, which requires the pro-mitophagic protein Atg32, is strongly reduced in cells lacking Egd1, a beta subunit of the nascent polypeptide-associated complex acting in cytosolic ribosome attachment and protein targeting to mitochondria. By contrast, loss of the sole alpha subunit Egd2 or the beta subunit paralogue Btt1 led to only a partial or slight reduction in mitophagy. We also found that phosphorylation of Atg32, a crucial step for priming mitophagy, is decreased in the absence of Egd1. Forced Atg32 hyperphosphorylation almost completely restored mitophagy in *egd1*-null cells. Together, we propose that Egd1 acts in Atg32 phosphorylation to facilitate mitophagy.

## Introduction

Mitochondria are essential organelles in most of eukaryotes, not only generating ATP but also participating in manifold processes including apoptosis, phospholipid metabolism, innate immunity, and cell differentiation^1–5^. Since mitochondrial dysfunction disrupts cellular homeostasis and causes diverse human diseases^6^, it is important for cells to remove malfunctioned mitochondria to maintain mitochondrial quality. One of the cellular pathways that can mediate mitochondrial degradation is mitophagy, an evolutionarily conserved catabolic process that drives selective removal of mitochondria via autophagy^7^. Under mitophagy-inducing conditions, mitochondria are enclosed by newly generated structures called isolation membranes that ultimately form autophagosomes, double membrane-bound vesicles^8,9^. Then, autophagosomes containing mitochondria fuse with lysosomes (or vacuoles in yeast), and the cargoes are subjected to hydrolytic degradation^8,9^. Recent studies using mammalian cells and mice have explored the physiological and pathophysiological functions of mitophagy and suggested that mitophagy acts in differentiation, cancer, neurodegeneration, and immune response^10–13^.

In the budding yeast *Saccharomyces cerevisiae*, mitophagy is induced by prolonged respiration to the stationary phase or a shift from fermentation or respiration to nitrogen starvation^14^. Mitophagy under these conditions is almost completely blocked by loss of Autophagy-related protein 32 (Atg32), a single-pass membrane protein consisting of 529 amino acid residues anchored on the surface of mitochondria, while other autophagy-related pathways are barely affected under the same conditions, indicating a specific role of Atg32 in mitochondrial degradation^15,16^. The Atg32(1–388) cytosolic domain is essential for mitophagy, as it has motifs to bind Atg8, a ubiquitin-like protein conjugated to the phospholipid phosphatidylethanolamine and localized to autophagosomes, and Atg11, a scaffold protein for assembly of core Atg proteins to generate autophagosomes containing selectively sequestered cargoes^15–18^. Disruption of Atg32–Atg8/11 interactions leads to suppression of mitophagy, supporting the idea that these three Atg proteins form a mitophagy initiation complex^17,18^. Notably, the Atg11-interaction motif of Atg32 has sequence similarity to those of other selective autophagy-promoting proteins^19^. Atg32(200–341), termed the pseudo-receiver (PsR) domain, is also thought to be critical for Atg32–Atg11 interactions and mitophagy activity during nitrogen starvation^20^. Moreover, transcriptional and post-translational regulations of Atg32 play roles in priming degradation of mitochondria, as Atg32 expression and phosphorylation are significantly induced under respiratory conditions, which may be activated by oxidative stress^15,17,18,21–25^.

Our previous genome-wide screen has revealed that mitophagy is reduced in cells lacking Egd1, an evolutionarily conserved subunit of the nascent polypeptide-associated complex (NAC)^15^. NAC serves as a peripheral component of cytoplasmic ribosomes and interacts with nascent polypeptide chains emerging from the ribosome^26^. In *S. cerevisiae*, three NAC subunits, α, β_1_, and β_3_, which are encoded by the *EGD2*, *EGD1*, and *BTT1* genes, respectively, form two distinct, heterodimeric complexes, αβ_1_ (Egd2/Egd1) and αβ_3_ (Egd2/Btt1)^27^. Loss of Egd2 causes partial defects in mitochondrial protein import^28^. In addition, cells lacking both Egd1 and Egd2 produce polypeptides normally, but have fewer ribosomes associated with the mitochondrial surface^29^. Consistent with these findings, Egd1 has been suggested to interact with Om14, a multi-pass mitochondrial outer membrane protein whose loss leads to a reduction in protein import into mitochondria in vitro^30^. Currently, it remains unclear how Egd1 acts in mitophagy.

In this study, we demonstrate that cells lacking Egd1 are strongly defective in mitophagy, whereas they exhibit a partial or slight reduction in other autophagy-related processes. Loss of Egd2 or Btt1 leads to partial or slight defects in mitophagy, supporting the idea that Egd1 functions in degradation of mitochondria independently of other NAC subunits. Atg32 phosphorylation is decreased in the absence of Egd1, and its hyperphosphorylation in *egd1*-null cells restores mitophagy at near wild-type levels. Surprisingly, Egd1 is dispensable for Atg32–Atg8/11 interactions, raising the possibility that Egd1 acts in unknown event(s) downstream of mitophagy-initiation complex assembly. Collectively, our data suggest that Egd1 contributes to mitophagy via its action in Atg32 phosphorylation.

## Results

### Transport of mitochondria to the vacuole is strongly suppressed in *egd1*-null cells

**Figure 1:**
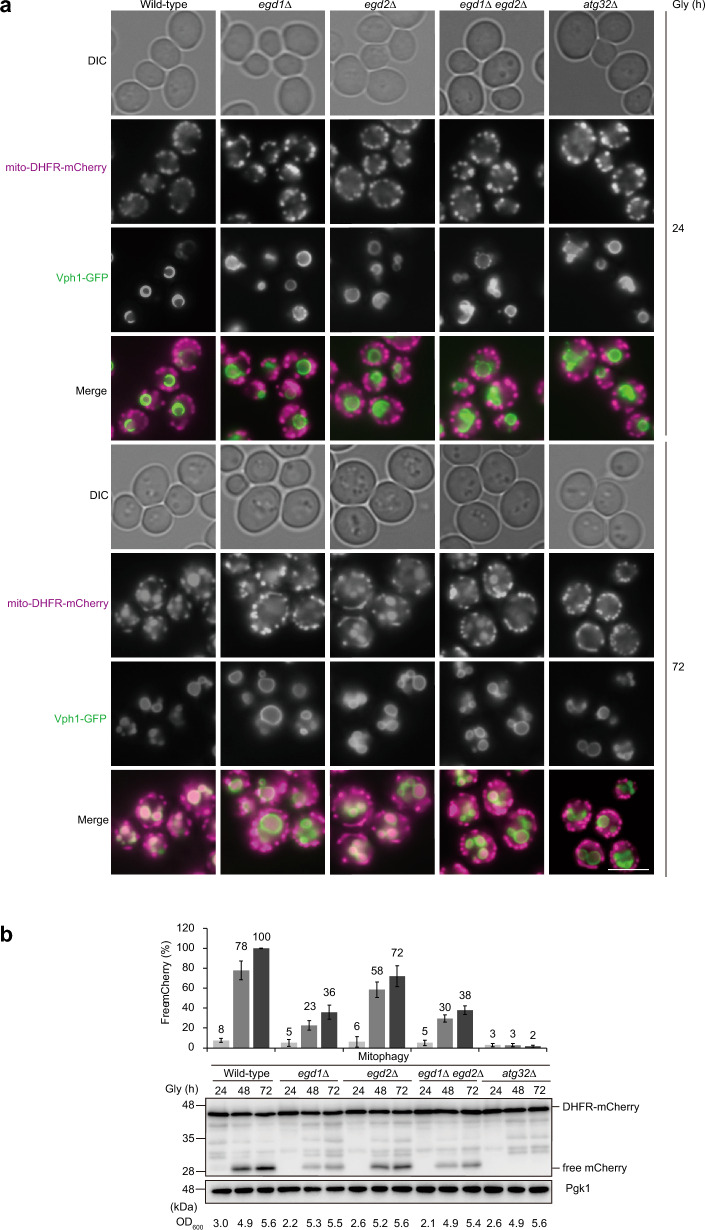
Mitophagy is strongly suppressed in cells lacking Egd1. (**a**) Representative images of mitochondrial mCherry and vacuolar GFP patterns under respiratory conditions. Wild-type, *egd1*∆, *egd2*∆, *egd1*∆ *egd2*∆, and *atg32*∆ cells expressing mito-DHFR-mCherry and Vph1-GFP grown to mid log phase in dextrose medium were cultured in glycerol medium (Gly) for 24 h and 72 h, and observed under a fluorescence microscope. Scale bar, 5 µm*.* DIC, differential interference contrast. (**b**) Wild-type, *egd1*∆, *egd2*∆, *egd1*∆ *egd2*∆, and *atg32*∆ cells expressing mito-DHFR-mCherry were grown for the indicated time points and OD_600_ values in glycerol medium (Gly) and subjected to western blotting. Generation of free mCherry indicates transport of mitochondria to the vacuole. Pgk1 was monitored as a loading control. The amounts of free mCherry in cells under respiratory conditions for 24 h, 48 h, and 72 h were quantified in three experiments. The signal intensity value of free mCherry in wild-type cells at the 72 h time point was set to 100%. Data represent the averages of all experiments, with bars indicating standard deviations.

To ask if Egd1 contributes to mitophagy in cooperation with Egd2, we first examined *egd1-*, *egd2-*, and *egd1/2-*null cells by fluorescence microscopy. Mitochondria and vacuoles were visualized using mito-DHFR-mCherry, a mitochondrial matrix-targeted mitophagy probe, and Vph1-GFP, a vacuole-anchored marker, respectively, to monitor transport of mitochondria to the vacuole^31^. Cells were grown in a medium containing glycerol as a non-fermentable carbon source and observed under a fluorescence microscope. Consistent with our previous observations^15^, mitochondrial mCherry signals were barely overlapped with vacuolar GFP signals in wild-type, *egd1-*, *egd2-*, and *egd1/2-*null cells at the 24 h time point (log phase) (Fig. 1a). By contrast, mCherry and GFP signals were often overlapped with each other in wild-type and *egd2*-null cells at the 72 h time point (stationary phase), indicating transport of mitochondria to the vacuole (note that mCherry is quite resistant against vacuolar proteases) (Fig. 1a). Under the same conditions, mCherry-GFP overlaps were strongly decreased in cells lacking Egd1 and Egd1/2.

To quantitatively evaluate mitophagy, we investigated *egd1-*, *egd2-*, and *egd1/2-*null cells by western blotting with anti-mCherry antibodies. When mitochondria are transported to the vacuole, mito-DHFR-mCherry is processed to generate free mCherry, thereby allowing us to semi-quantify mitophagy^31^. Consistent with the microscopic observations, free mCherry levels were reduced in *egd1-*, *egd2-*, and *egd1/2-*null cells (36%, 72%, and 38%, respectively, compared to wild-type cells at the 72 h time point (Fig. 1b). By contrast, loss of Btt1, another NAC β subunit, led to only a minor effect on mitophagy (Fig. S1a,b). These results suggest that Egd1 acts in mitophagy independently of Egd2 and Btt1.

### Other autophagy-related pathways are only partially or slightly affected in the absence of Egd1/2

**Figure 2:**
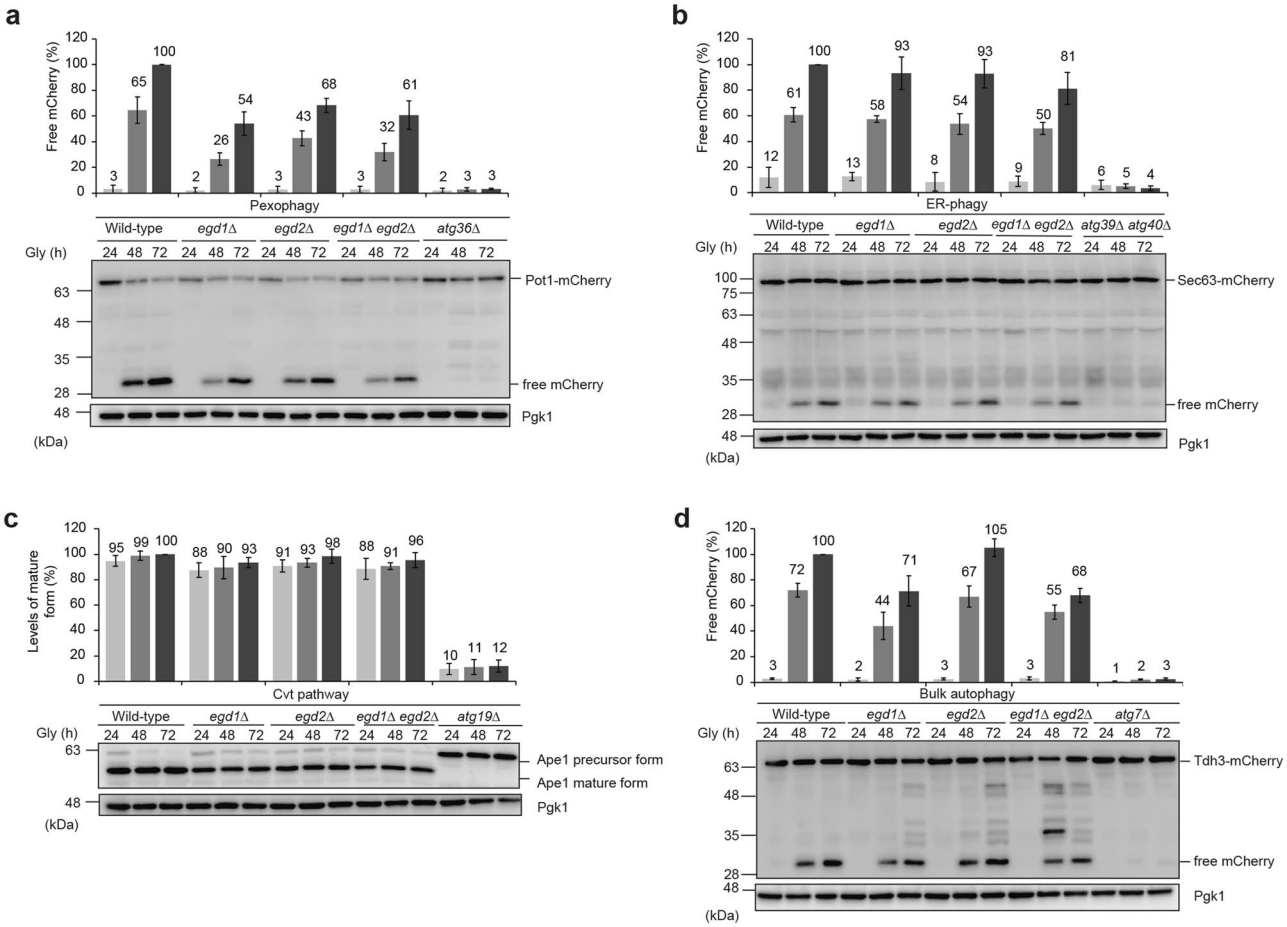
Other autophagy-related pathways are only partially or slightly reduced in the absence of Egd1. (**a**) Wild-type, *egd1*∆, *egd2*∆, *egd1*∆ *egd2*∆, and *atg36*∆ cells expressing Pot1-mCherry (a peroxisome marker) were grown in glycerol medium (Gly), collected at the indicated time points, and subjected to western blotting. The amounts of free mCherry in cells under respiratory conditions for 24 h, 48 h, and 72 h were quantified in three experiments. The signal intensity value of free mCherry in wild-type cells at the 72 h time point was set to 100%. Data represent the averages of all experiments, with bars indicating standard deviations. Pgk1 was monitored as a loading control. (**b**) Wild-type, *egd1*∆, *egd2*∆, *egd1*∆ *egd2*∆, and *atg39*∆ *atg40*∆ cells expressing Sec63-mCherry (an ER marker) were analyzed as (**a**). (**c**) Wild-type, *egd1*∆, *egd2*∆, *egd1*∆ *egd2*∆, and *atg19*∆ cells expressing mito-DHFR-mCherry were analyzed as (**a**). (**d**) Wild-type, *egd1*∆, *egd2*∆, *egd1*∆ *egd2*∆, and *atg7*∆ cells expressing Tdh3-mCherry (a cytosol marker) were analyzed as (**a**).

Given the fact that prolonged respiration also induces autophagy-dependent breakdown for other organelles and proteins^32^, we asked if Egd1 and Egd2 serve to promote those catabolic events in cells under respiratory conditions. First, we examined selective autophagic degradation of peroxisomes (pexophagy) using Pot1-mCherry, a marker localized in the peroxisomal matrix. As described previously, Pot1-mCherry is transported to the vacuole in a manner dependent on Atg36, a protein essential for pexophagy^33^ and processed to become free mCherry. We performed western blotting and detected a decrease in free mCherry signals to 54%, 68%, and 61% in *egd1-*, *egd2-*, and *egd1/2-*null cells, respectively, compared to wild-type cells at the 72 h time point (Fig. 2a). These results raise the possibility that Egd1/2 may act together in pexophagy.

Second, we examined endoplasmic reticulum-specific autophagy (ER-phagy) using Sec63-mCherry under respiratory conditions. Similar to the mitophagy marker mito-DHFR-mCherry, this ER membrane-anchored probe is transported to the vacuole and processed to generate free mCherry in a manner dependent on Atg39 and Atg40^34^, two integral membrane proteins required for ER-phagy. We found that free mCherry signals were reduced to 93%, 93%, and 81% in *egd1-*, *egd2-*, and *egd1/2-*null cells, respectively, compared to wild-type cells at the 72 h time point (Fig. 2b). Thus, it seems likely that cells undergo ER-phagy almost independently of Egd1/2.

Third, we examined the cytoplasm-to-vacuole targeting (Cvt) pathway, an autophagy-related process selective for vacuolar enzymes such as the aminopeptidase Ape1^35^. A precursor form of this hydrolase is constitutively synthesized in the cytosol, transported to the vacuole in a manner dependent on Atg19^35^, an adaptor protein required for the Cvt pathway, and processed to become a mature form. During prolonged respiratory growth, *egd1-*, *egd2-*, and *egd1/2-*null cells exhibited efficient, wild-type-like Ape1 maturation (93%, 98%, and 96%, respectively, compared to wild-type cells at the 72 h time point) (Fig. 2c), indicating that Egd1/2 is mostly dispensable for the Cvt pathway.

Finally, we examined bulk autophagy that was also promoted in cells under respiratory conditions. Tdh3-mCherry, a cytoplasmic marker that is transported to the vacuole in an Atg7-dependent fashion and processed to become free mCherry, was used to investigate bulk autophagy in cells lacking Egd1, Egd2, and Egd1/2. Free mCherry signals were partially or slightly reduced in *egd1-*, *egd2-*, and *egd1/2-*null cells (71%, 105%, and 68%, respectively, compared to wild-type cells at the 72 h time point) (Fig. 2d), raising the possibility that Egd1 may contribute to efficient bulk autophagy independently of Egd2.

### Atg32 phosphorylation is reduced in cells lacking Egd1

In cells under respiratory conditions, the pro-mitophagic membrane protein Atg32 is upregulated in response to oxidative stress, anchored to the surface of mitochondria, and activated via phosphorylation. Then, Atg32 interacts with Atg8 and Atg11, forming a ternary complex to recruit other core Atg proteins required for autophagosome formation, thereby promoting mitophagy (Fig. S2a). Accordingly, we investigated whether loss of Egd1 leads to an alteration in Atg32 expression, mitochondrial localization, phosphorylation, or complex assembly.

**Figure 3:**
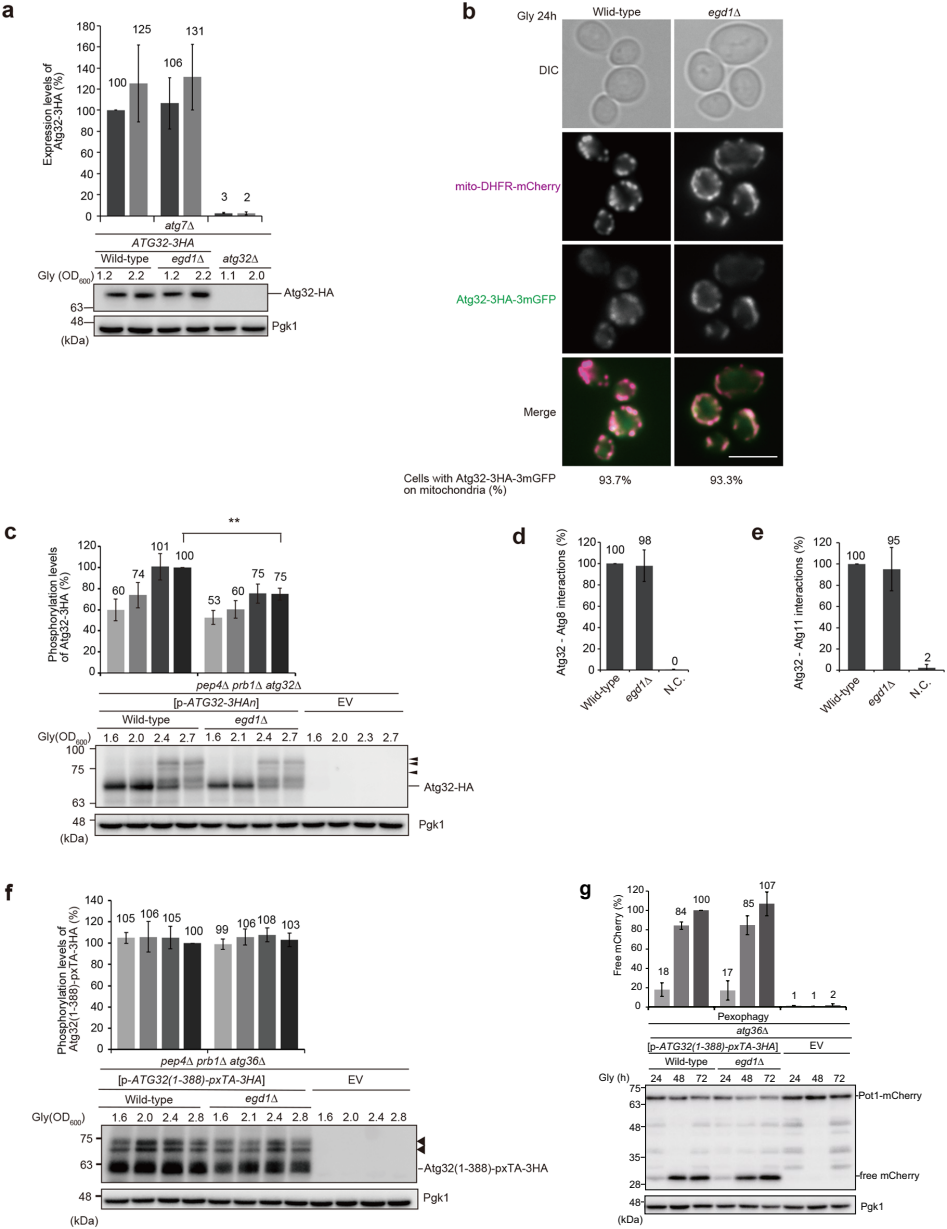
Loss of Egd1 leads to a decrease in Atg32 phosphorylation. (**a**) Wild-type, *egd1*∆, and *atg32*∆ cells expressing Atg32-3HA were grown in glycerol medium (Gly), collected at the indicated OD_600_ points, and subjected to western blotting. All strains are derivatives lacking Atg7, a protein essential for all autophagy-related processes, to avoid degradation of Atg32-3HA via mitophagy. Atg32-3HA signals normalized with Pgk1 (loading control) signals were quantified more than three times in independent experiments. The expression level of Atg32-3HA in wild-type cells at the OD_600_ = 1.2 point was set to 100%. Data represent the averages of all experiments, with bars indicating standard deviations. (**b**) Wild-type and *egd1*∆ cells expressing mito-DHFR-mCherry and Atg32-3HA-3mGFP were grown in glycerol medium for 24 h and observed under a fluorescence microscope. Cells (n = 100) with mitochondria-localized Atg32-3HA-3mGFP signals were quantified in more than three experiments, and the average percentages were indicated on the bottom side of image panels. Scale bar, 5 µm. (**c**) Wild-type and *egd1*∆ cells transformed with a plasmid encoding Atg32-3HA (p-*ATG32-3HA*), or an empty vector (EV) were grown in non-fermentable glycerol medium (Gly), collected at the indicated OD_600_ points, and subjected to western blotting. All strains are *pep4 prb1 atg32* triple-null derivatives defective in intravacuolar degradation. Arrowheads indicate putative phosphorylated Atg32. Phosphorylated Atg32-3HA signals normalized with all Atg32-3HA signals were quantified more than three times in independent experiments. The phosphorylation level of Atg32-3HA in wild-type cells at the OD_600_ = 2.7 point was set to 100%. Data represent the averages of all experiments, with bars indicating standard deviations. **P < 0.01 (unpaired two-tailed Student’s t-test). (**d**, **e**) Wild-type and *egd1*∆ cells expressing Atg32-3HA-3mGFP-3FLAG-LgBiT and SmBiT-3FLAG-8His-Atg8 or Atg11-HA-SmBiT, or wild-type cells expressing Atg32 and Atg11 (negative control, N.C.) were grown in glycerol medium (Gly), collected at the OD_600_ = 1.4 point, incubated with substrates, and subjected to measurements of GFP and luminescent signals in more than three experiments using a microplate reader. (**f**) Wild-type and *egd1*∆ cells transformed with a plasmid encoding the Atg32 cytoplasmic domain anchored to the peroxisome (p-*Atg32(1–388)-pxTA-3HA*), or an empty vector (EV) were grown in non-fermentable glycerol medium (Gly), collected at the indicated OD_600_ points, and subjected to western blotting. All strains are *pep4 prb1 atg36* triple-null derivatives defective in intravacuolar degradation and endogenous pexophagy. Arrowheads indicate putative phosphorylated protein bands. Phosphorylated Atg32(1–388)-pxTA-3HA signals normalized with all Atg32(1–388)-pxTA-3HA signals were quantified more than three times in independent experiments. The phosphorylation level of Atg32(1–388)-pxTA-3HA in wild-type cells at the OD_600_ = 2.8 point was set to 100%. Data represent the averages of all experiments, with bars indicating standard deviations. (**g**) Wild-type and *egd1*∆ cells transformed with p-*Atg32(1–388)-pxTA-3HA*, or an empty vector (EV) were grown in non-fermentable glycerol medium (Gly), collected at the indicated time points, and subjected to western blotting. All strains are *atg36*-null derivatives expressing Pot1-mCherry (a peroxisome marker). The amounts of free mCherry in cells under respiratory conditions for 24 h, 48 h, and 72 h were quantified in three experiments. The signal intensity value of free mCherry in wild-type cells at the 72 h time point was set to 100%. Data represent the averages of all experiments, with bars indicating standard deviations.

First, to analyze Atg32 expression, cells containing the chromosomally HA-tagged *ATG32* gene were subjected to western blotting. Samples were prepared from cells at the OD_600_ value points, as Atg32 expression and the subsequent events change in correlation with the growth phase rather than the culture time^23^. We found that loss of Egd1 did not significantly alter Atg32-3HA protein levels in cells under respiratory conditions (Fig. 3a).

Second, fluorescence microscopy was performed to observe cells expressing Atg32-3HA-3mGFP and the mitochondrial marker mito-DHFR-mCherry. We found that Atg32-3HA-3mGFP mostly co-localized with mito-DHFR-mCherry in both wild-type and *egd1*-null cells, suggesting that Egd1 is dispensable for Atg32 mitochondrial targeting (Fig. 3b).

Third, we asked whether Atg32 phosphorylation upon mitophagy induction is affected by loss of Egd1. In wild-type cells, phosphorylated Atg32 molecules appeared at mid- to late-log phase of respiratory growth, which can be detected as multiple upper bands by SDS-PAGE^17,18^ (Fig. 3c). By contrast, Atg32 phosphorylation was reduced in *egd1-null* cells (75% compared to wild-type cells at the OD_600_ = 2.7 point) (Fig. 3c). Under the same conditions, we found that Atg32 phosphorylation was hardly affected in cells lacking Egd2 or Btt1 (Fig. S2e). These results raise the possibility that Egd1 may contribute to mitophagy via its action on Atg32 phosphorylation.

Fourth, Atg32–Atg8 and Atg32–Atg11 interactions, key events for mitophagy-initiation complex assembly, were analyzed using NanoLuc Binary Technology (NanoBiT), a split luciferase-based reporter system composed of the large BiT (LgBiT; 18 kDa) subunit and the small BiT (SmBiT; 11 amino acid residues) peptide^36^. To this end, we generated *egd1*-null cells expressing LgBiT-tagged Atg32 and SmBiT-tagged Atg11 or Atg8, grew them to mid-log phase under respiratory conditions, and subjected them to a bioluminescence assay. When Atg32-LgBiT forms a complex with SmBiT-Atg8 or Atg11-SmBiT, the two split NanoLuc parts LgBiT and SmBiT are brought into close proximity, resulting in reversible reconstitution of an active luciferase that generates bright luminescent signals in the presence of its substrate furimazine^36^ (Fig. S2b). We found that both Atg32–Atg11 and Atg32–Atg8 interactions are not significantly altered in the absence of Egd1 (Fig. 3d,e). In addition, Atg8 and Atg11 protein levels were hardly altered in cells lacking Egd1 (Fig. S2c,d). Thus, it seems likely that loss of Egd1 leads to a reduction in Atg32 phosphorylation, thereby suppressing mitophagy but without compromising the mitophagy-initiation complex assembly.

We have previously demonstrated that artificially peroxisome-anchored cytosolic domain of Atg32 is phosphorylated and activated to drive pexophagy under respiratory conditions^17^. Accordingly, we asked if phosphorylation of this Atg32 variant, Atg32(1–388)-pxTA-3HA, and the mitophagic factor-mediated pexophagy are altered in the absence of Egd1. Surprisingly, Atg32 (1–388)-pxTA-3HA phosphorylation was hardly reduced in cells lacking Egd1 (Fig. 3f). Consistent with these data, pexophagy was merely decreased in *egd1*-null cells expressing Atg32(1–388)-pxTA-3HA (Fig. 3g). These results suggest that Egd1 acts in Atg32 phosphorylation and contributes to Atg32-mediated autophagic degradation when Atg32 is anchored to mitochondria.

### Hyperphosphorylation of Atg32 restores mitophagy in *egd1*-null cells

**Figure 4:**
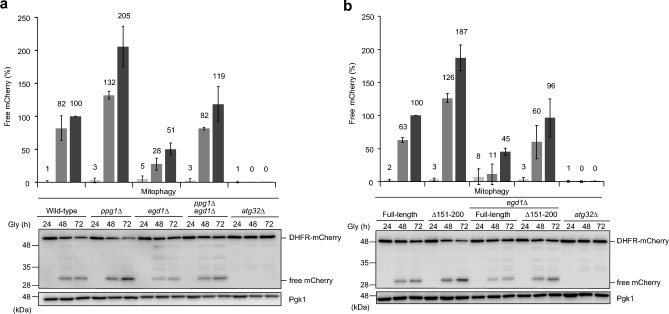
Mitophagic deficiencies in *egd1*-null cells can be rescued by Atg32 hyperphosphorylation. (**a**) Wild-type, *ppg1*∆, *egd1*∆, *egd1*∆ *ppg1*∆, and *atg32*∆ cells expressing mito-DHFR-mCherry were grown in glycerol medium (Gly), collected at the indicated time points, and subjected to western blotting. The amounts of free mCherry in cells under respiratory conditions for 24 h, 48 h, and 72 h were quantified in three experiments. The signal intensity value of free mCherry in wild-type cells at the 72 h time point was set to 100%. Data represent the averages of all experiments, with bars indicating standard deviations. (**b**) Wild-type and *egd1*∆ cells expressing Atg32-3HA (Full-length) or an Atg32 deletion mutants fused with 3HA (∆151–200), or *atg32*-null cells (negative control) were analyzed as (**a**). All strains are derivatives expressing mito-DHFR-mCherry.

It has been shown that the Ppg1-Far phosphatase complex mediates dephosphorylation of Atg32 and negatively regulates mitophagy^37^ (Fig. S3a). Accordingly, we speculated that enhanced phosphorylation of Atg32 could rescue mitophagy defects in cells lacking Egd1. To test this idea, we performed mitophagy assays and found that free mCherry levels in *ppg1 egd1* double-null cells were increased to 119% compared to wild-type cells at the 72 h time point (Fig. 4a). These results indicate that loss of Ppg1 restores Atg32 phosphorylation, thereby promoting mitophagy in cells lacking Egd1.

Next, we sought to investigate whether an acceleration in Atg32 phosphorylation can rescue mitophagy defects in *egd1*-null cells without compromising Ppg1 function. To this end, cells expressing Atg32(∆151–200)-3HA, an Atg32 variant lacking the amino acid residues 151–200 required for Ppg1-Far binding, were subjected to mitophagy assays. Expression of this truncated protein has been shown to increase Atg32 phosphorylation and accelerate mitophagy even in wild-type cells^37^ (Fig. 4b). We found that mitophagy was restored in *egd1-*null cells expressing Atg32(∆151–200)-3HA (96% compared to wild-type cells expressing Atg32-3HA at the 72 h time point) (Fig. 4b).

To exclude the possibility that these recoveries are attributed to an increase in Atg32 expression, Atg32-3HA and Atg32(∆151–200)-3HA protein levels were examined in cells under respiratory conditions. Loss of Ppg1 or deletion of the Atg32 amino acid residues 151–200 did not significantly alter Atg32 protein levels in the presence or absence of Egd1 (Fig. S3b,c). By contrast, Atg32 phosphorylation levels in cells lacking Egd1 were indeed increased by loss of Ppg1 (125% compared to wild-type cells at the OD_600_ = 2.8 point) (Fig. S3d), further supporting the idea that mitophagy recoveries in this double deletion mutant result from an increase in Atg32 phosphorylation.

As the mitochondria-targeted Ppg1-Far complex negatively regulates Atg32 phosphorylation^38,39^, it remains possible that loss of Egd1 leads to Ppg1-Far mitochondrial localization, thereby suppressing mitophagy. To test this idea, we performed fluorescence microscopy for cells expressing a 3 × GFP-tagged version of Far8, a component of the Ppg1-Far complex essential for Ppg1 phosphatase activity, and the ER marker Sec63-mCherry or the mitochondrial marker mito-DHFR-mCherry. At mid-log phase during respiratory growth, Far8-3 × GFP predominantly co-localized with Sec63-mCherry in both wild-type and *egd1*-null cells (59% and 69% of the total population, respectively) (Fig. S3e). Under the same conditions, cells lacking Get3, a cytosolic ATPase acting in ER targeting of the Ppg1-Far complex^39^, exhibited Far8-3 × GFP mostly co-localized with mito-DHFR-mCherry (79% of the total population) (Fig. S3e). These observations suggest that localization of the Ppg1-Far complex is not significantly altered in the absence of Egd1.

Collectively, these results are consistent with the idea that a Ppg1-Far-independent decrease in Atg32 phosphorylation is the primary cause of mitophagy deficiencies in cells lacking Egd1.

## Discussion

In this study, we demonstrate that the NAC subunit Egd1 acts in respiration-induced mitophagy independently of its partner subunit Egd2 (Fig. 1a,b). Under the same conditions, other selective and bulk autophagy pathways are only slightly or partially affected by loss of Egd1 and/or Egd2 (Fig. 2a–d). Thus, it is conceivable that the NAC function may not directly be involved in mitophagy, and that Egd1 may participate solely and specifically in degradation of mitochondria.

How does Egd1 function in mitophagy? Our data reveal that Atg32 expression (Fig. 3a), mitochondrial localization (Fig. 3b), and complex assembly (Atg32–Atg8/11 interactions) (Fig. 3d,e) are not significantly altered in the absence of Egd1. Remarkably, Atg32 phosphorylation is reduced in cells lacking Egd1 under respiratory conditions (Fig. 3c). These findings imply that Egd1-dependent Atg32 phosphorylation is not required for Atg32-Atg8/11 interactions but rather critical for previously unappreciated process(es) downstream of mitophagy-initiation complex assembly to promote mitophagy. It should also be noted that Egd1 is dispensable for pexophagy in cells expressing peroxisome-anchored Atg32 variant (Fig. 3g), despite that the Atg36-mediated pexophagy is partially impaired in the absence of Egd1 (Fig. 2a). Thus, we are in favor of the idea that Egd1 function for Atg32 is limited to mitochondria in a manner distinct from that for Atg36-mediated pexophagy.

How Egd1 contributes to Atg32 phosphorylation is currently unknown. Strikingly, mitophagy deficiencies in *egd1*-null cells can mostly be rescued by Ppg1-related hyperphosphorylation of Atg32 (Fig. 4a,b). These results raise the possibility that loss of Egd1 might lead to an alteration in activity and/or localization of the Ppg1-Far complex, thereby suppressing Atg32 phosphorylation and mitophagy. It is, however, unlikely to be the case, since Atg32–Atg11 interactions, which are negatively regulated by the Ppg1-Far complex, do not seem to be affected in cells lacking Egd1 (Fig. 3d,e). Further studies are needed to clarify how Egd1 regulates Atg32 phosphorylation and how Egd1-dependent Atg32 phosphorylation promotes mitochondrial clearance.

## Methods

### Yeast strains and growth conditions

Yeast strains used in this study are listed in Supplementary Tables 1 and 2. Standard genetic and molecular biology methods were used. Yeast cells were incubated in YPD medium (1% yeast extract, 2% peptone, and 2% dextrose), synthetic medium (0.17% yeast nitrogen base without amino acids and ammonium sulfate, 0.5% ammonium sulfate) with 0.5% casamino acids containing 2% dextrose (SDCA), or 0.1% dextrose plus 3% glycerol (SDGCA), supplemented with necessary amino acids. For all assays under respiratory conditions, cells pre-grown to mid-log phase in SDCA were transferred to SDGCA and incubated at 30 °C.

### Fluorescence microscopy

Live yeast cells expressing Vph1-GFP, Atg32-3HA-3mGFP, or Far8-3×GFP, and mito-DHFR-mCherry or Sec63-mCherry were observed using structured illumination microscopy (SIM). Differential interference contrast (DIC) and fluorescence images were obtained under a Pulse-SIM BZ-X800 microscope (Keyence) equipped with a 100 × objective lens (CFI Apochromat TIRF 100XC Oil, NA: 1.49; Nikon), filter sets for GFP and mCherry (BZ-X filter GFP and BZ-X filter TRITC, respectively; Keyence) and an optical sectioning module (BZ-H4XF; Keyence).

### Protein extraction

1.0 OD_600_ units of cells were collected by centrifugation at 7900×*g* for 30 s, and the supernatant was discarded using a micropipette. The cell pellet was resuspended in 100 μL of 0.1N NaOH and incubated for 5 min at room temperature to enhance the permeability of the cell wall. The cells were subjected to centrifugation (17,000×*g*) at room temperature for 3 min, the pellet was resuspended in 50 μL SDS-sample buffer (60 mM Tris–HCl (pH 6.8), 5% glycerol, 2% SDS, 20 mM DTT, 0.00025% bromophenol blue). The samples were boiled for 3 min at 100 °C, kept on ice for 1 min, and subjected to centrifugation (17,000×*g*) at room temperature for 3 min. Supernatants were transferred to new tubes and stored at − 80 °C.

### Western blotting

Samples corresponding to 0.1–0.4 OD_600_ units of cells were separated by SDS-PAGE followed by western blotting and immunodecoration with primary antibodies raised against mCherry (1:2000, Abcam ab125096), Pgk1 (1:10,000, Abcam, ab113687), HA (1:5000, 16B12, Covance), Ape1 (1:5000), Atg8 (1:2000) (gifts from Dr. Hitoshi Nakatogawa, Tokyo Institute of Technology, Japan), and Atg11 (1:1000) (a gift from Dr. Hayashi Yamamoto, Nippon Medical School, Japan). After treatment with the secondary antibodies, horseradish peroxidase (HRP)-conjugated rabbit anti-mouse IgG (H + L) for mCherry, HA, Pgk1, and goat anti-rabbit IgG (H + L) for Ape1, Atg8 and Atg11 (1:10,000, Jackson ImmunoResearch 315-035-003 and 111-035-003, respectively) followed by the enhanced chemiluminescence reagent Western Lightning Plus-ECL (PerkinElmer, 203-19151) or Chemi-Lumi One L (nacalai tesque, 07880-70), proteins were detected using a luminescent image analyzer (FUSION Solo S; VILBER). Quantification of the signals was performed using FUSION Solo S (VILBER).

### Bioluminescence assay for protein–protein interactions

For quantitative analysis of Atg32–Atg8/11 interactions utilizing NanoBiT (Promega), Atg32, fused internally at the 179–180 a.a. to 3 × HA, 3 × mGFP, 3 × FLAG, and LgBiT (Atg32-3HA-3mGFP-3FLAG-LgBiT), and Atg8 or Atg11, fused N- or C-terminally, respectively, to SmBiT, 3 × FLAG, and 8 × His (SmBiT-3FLAG-8His-Atg8), or HA and SmBiT (Atg11-HA-SmBiT), were endogenously expressed from their chromosomal loci (constructed by Ritsu Shibata and Yang Liu, Osaka University, Japan). To evaluate Atg32–Atg8/11 interactions in vivo, 1.0 OD_600_ units of cells grown in SDGCA were harvested at the OD_600_ = 1.4 point and rinsed with 1 mL of PBS. Cells were then resuspended in 40 µL of PBS and introduced to a 96-well plate. The detection reagent was prepared by diluting the Nano-Glo Live Cell Substrate (Promega, 0000360026) 1:10 with the Nano-Glo LCS Dilution Buffer (Promega, 0000333050) to create the Nano-Glo Live Cell Reagent. A volume of 10 µL diluted detection reagent was added to the 96-well plate and mixed with the cells, followed by incubation at 30 °C for 1 h. The resultant luminescent signals were detected using the Fluoroskan Ascent FL microplate reader (Thermo Fisher Scientific) with an exposure time of 1 s. To detect GFP fluorescent signals derived from Atg32, 0.5 OD_600_ units of cells were simultaneously collected, resuspended in 100 µL of PBS, and loaded onto the 96-well plate. To normalize the luminescence intensity, GFP signals were measured by the Fluoroskan Ascent FL microplate reader (Thermo Fisher Scientific) with an excitation at 485 nm, an emission at 538 nm, and an exposure time of 1 s.

### Statistical analysis

Results are presented as means including standard deviation (SD). Statistical tests were performed using t-test with Microsoft Excel (Microsoft Office for Mac).

### Supplementary Information

Supplementary Information.

## Data Availability

The datasets generated and analyzed during the current study are available from the corresponding author on reasonable request.
