# Author Correction: The nascent polypeptide-associated complex subunit Egd1 is required for efficient selective mitochondrial degradation in budding yeast

**DOI:** 10.1038/s41598-024-59028-0

**Published:** 2024-04-11

**Authors:** Yuan Tian, Koji Okamoto

**Affiliations:** Laboratory of Mitochondrial Dynamics, Graduate School of Frontier Biosciences, Osaka University, Suita, Osaka 565-0871 Japan

Correction to: *Scientific Reports* 10.1038/s41598-023-50245-7, published online 04 January 2024

The original version of this Article contained an error in Figure 3 where the western blot decorated with the anti-Pgk1 antibody for loading control was incorrect in panel (g). The original Figure 3 and accompanying legend appear below.

**Figure 3:**
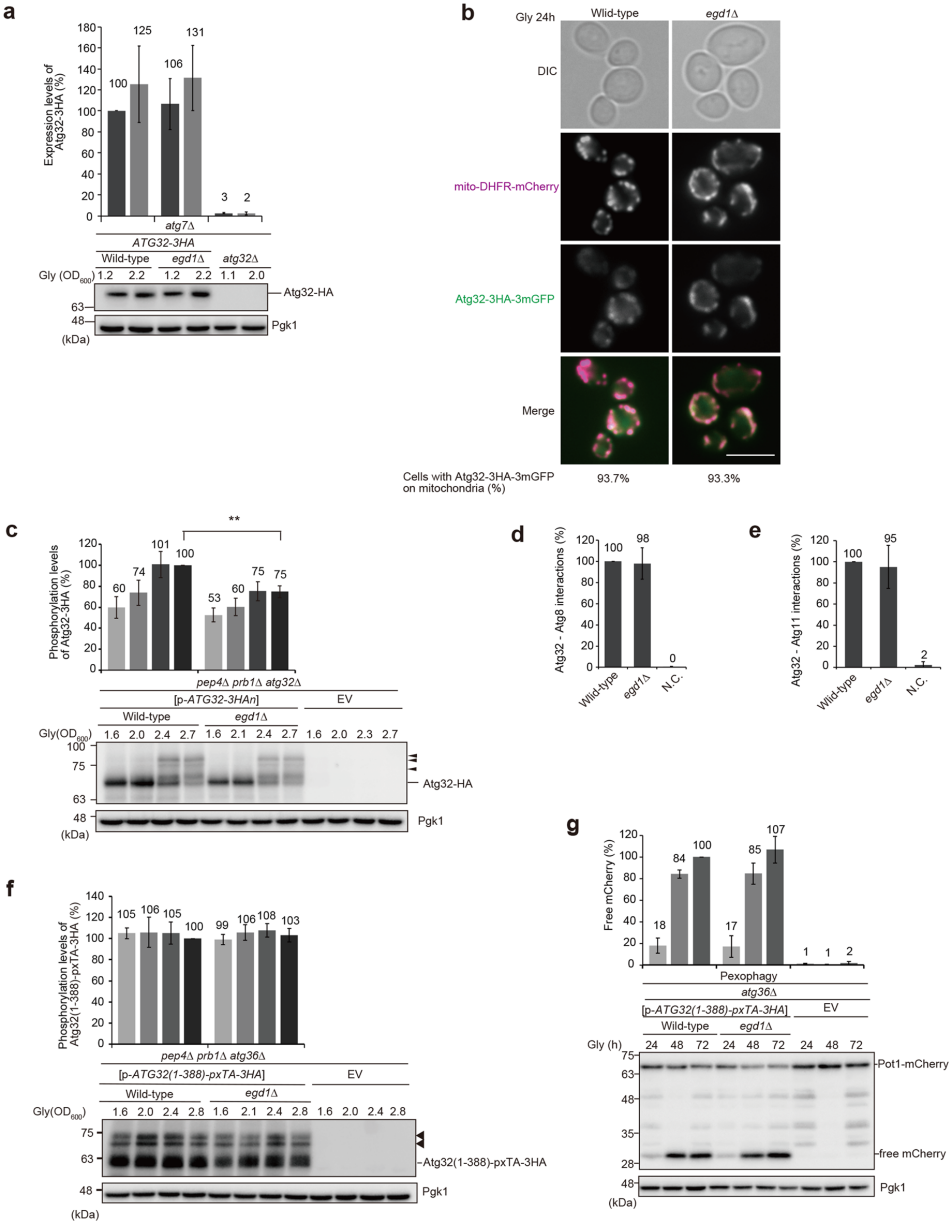
Loss of Egd1 leads to a decrease in Atg32 phosphorylation. (**a**) Wild-type, *egd1*∆, and *atg32*∆ cells expressing Atg32-3HA were grown in glycerol medium (Gly), collected at the indicated OD_600_ points, and subjected to western blotting. All strains are derivatives lacking Atg7, a protein essential for all autophagy-related processes, to avoid degradation of Atg32-3HA via mitophagy. Atg32-3HA signals normalized with Pgk1 (loading control) signals were quantified more than three times in independent experiments. The expression level of Atg32-3HA in wild-type cells at the OD_600_ = 1.2 point was set to 100%. Data represent the averages of all experiments, with bars indicating standard deviations. (**b**) Wild-type and *egd1*∆ cells expressing mito-DHFR-mCherry and Atg32-3HA-3mGFP were grown in glycerol medium for 24 h and observed under a fluorescence microscope. Cells (n = 100) with mitochondria-localized Atg32-3HA-3mGFP signals were quantified in more than three experiments, and the average percentages were indicated on the bottom side of image panels. Scale bar, 5 µm. (**c**) Wild-type and *egd1*∆ cells transformed with a plasmid encoding Atg32-3HA (p-*ATG32-3HA*), or an empty vector (EV) were grown in non-fermentable glycerol medium (Gly), collected at the indicated OD_600_ points, and subjected to western blotting. All strains are *pep4 prb1 atg32* triple-null derivatives defective in intravacuolar degradation. Arrowheads indicate putative phosphorylated Atg32. Phosphorylated Atg32-3HA signals normalized with all Atg32-3HA signals were quantified more than three times in independent experiments. The phosphorylation level of Atg32-3HA in wild-type cells at the OD_600_ = 2.7 point was set to 100%. Data represent the averages of all experiments, with bars indicating standard deviations. **P < 0.01 (unpaired two-tailed Student’s t-test). (**d**, **e**) Wild-type and *egd1*∆ cells expressing Atg32-3HA-3mGFP-3FLAG-LgBiT and SmBiT-3FLAG-8His-Atg8 or Atg11-HA-SmBiT, or wild-type cells expressing Atg32 and Atg11 (negative control, N.C.) were grown in glycerol medium (Gly), collected at the OD_600_ = 1.4 point, incubated with substrates, and subjected to measurements of GFP and luminescent signals in more than three experiments using a microplate reader. (**f**) Wild-type and *egd1*∆ cells transformed with a plasmid encoding the Atg32 cytoplasmic domain anchored to the peroxisome (p-*Atg32(1–388)-pxTA-3HA*), or an empty vector (EV) were grown in non-fermentable glycerol medium (Gly), collected at the indicated OD_600_ points, and subjected to western blotting. All strains are *pep4 prb1 atg36* triple-null derivatives defective in intravacuolar degradation and endogenous pexophagy. Arrowheads indicate putative phosphorylated protein bands. Phosphorylated Atg32(1–388)-pxTA-3HA signals normalized with all Atg32(1–388)-pxTA-3HA signals were quantified more than three times in independent experiments. The phosphorylation level of Atg32(1–388)-pxTA-3HA in wild-type cells at the OD_600_ = 2.8 point was set to 100%. Data represent the averages of all experiments, with bars indicating standard deviations. (**g**) Wild-type and *egd1*∆ cells transformed with p-*Atg32(1–388)-pxTA-3HA*, or an empty vector (EV) were grown in non-fermentable glycerol medium (Gly), collected at the indicated time points, and subjected to western blotting. All strains are *atg36*-null derivatives expressing Pot1-mCherry (a peroxisome marker). The amounts of free mCherry in cells under respiratory conditions for 24 h, 48 h, and 72 h were quantified in three experiments. The signal intensity value of free mCherry in wild-type cells at the 72 h time point was set to 100%. Data represent the averages of all experiments, with bars indicating standard deviations.

The original Article has been corrected.

