# Supplementary material for: The nascent polypeptide-associated complex subunit Egd1 is required for efficient selective mitochondrial degradation in budding yeast
Source: Sci Rep. 2024 Jan 4;14:546. doi: 10.1038/s41598-023-50245-7 (PMC10767044; doi:10.1038/s41598-023-50245-7)
Supplement: Supplementary file 1 — Supplementary Information. [file 41598_2023_50245_MOESM1_ESM.pdf]

The nascent polypeptide-associated complex subunit Egd1 is required for efficient selective mitochondrial degradation in budding yeast

Yuan Tian and Koji Okamoto*

Laboratory of Mitochondrial Dynamics, Graduate School of Frontier Biosciences, Osaka University, Suita, Osaka 565-0871, Japan

*Corresponding author. E-mail: okamoto.koji.fbs@osaka-u.ac.jp

Supplementary Figure legends

Supplementary Figure 1. (**a**) Representative images of mitochondrial mCherry and vacuolar GFP localizations under respiratory conditions. Wild-type, *egd1*Δ, *btt1*Δ, *egd1*Δ *btt1*Δ*,* and *atg32*Δ cells expressing mito-DHFR-mCherry and Vph1-GFP grown to mid log phase in dextrose medium were cultured in glycerol medium (Gly) for 24h and 72h, and observed under a fluorescence microscope. Scale bar, 5 μm. DIC, differential interference contrast. (**b**) Wild-type, *egd1*Δ, *btt1*Δ, *egd1*Δ *btt1*Δ*,* and *atg32*Δ cells expressing mito-DHFR-mCherry were grown for the indicated time points in glycerol medium (Gly) and subjected to western blotting. Generation of free mCherry indicates transport of mitochondria to the vacuole. Pgk1 was monitored as a loading control. The amounts of free mCherry in cells under respiratory conditions for 24 h, 48 h, and 72 h were quantified in three experiments. The signal intensity value of free mCherry in wild-type cells at the 72h time point was set to 100%. Data represent the averages of all experiments, with bars indicating standard deviations.

Supplementary Figure 2. (**a**) A model for initiation of Atg32-mediated mitophagy in buddding yeast. (**b**) A schematic diagram of the NanoBiT system applied to analyze Atg32-Atg11 and Atg32-Atg8 interactions. (**c**) Wild-type, *egd1*Δ, and *atg11*Δ cells were grown in glycerol medium (Gly), collected at the indicated OD_{600} points, and subjected to western blotting. All strains are *pep4*Δ *prb1*Δ derivatives defective in intravacuolar degradation. The amounts of Atg11 in cells at OD_{600} = 1.2 or 2.2 point were quantified. The signal intensity value of Atg11 in wild-type cells at OD_{600} = 2.2 point was set to 100%. (**d**) Wild-type, *egd1*Δ, *atg7*Δ, and *atg8*Δ cells were grown in glycerol medium (Gly), collected at the indicated OD_{600} points, and subjected to western blotting. All strains are *pep4*Δ *prb1*Δ derivatives defective in intravacuolar degradation. The amounts of Atg8 and Atg8-PE in cells at OD_{600} = 1.2 or 2.2 point were quantified. The signal intensity value of Atg8 and Atg8-PE in wild-type cells at OD_{600} = 2.2 point was set to 100%. (**e**) Wild-type, *egd2*Δ, and *btt1*Δ cells transformed with a plasmid encoding Atg32-3HA (p-*ATG32-3HA*), or an empty vector (EV) were grown in non-fermentable glycerol medium (Gly), collected at the indicated OD_{600} points, and subjected to western blotting. All strains are *pep4*Δ *prb1*Δ *atg32*Δ derivatives defective in intravacuolar degradation and mitophagy. Arrowheads indicate putative phosphorylated Atg32. Phosphorylated Atg32-3HA signals normalized with all Atg32-3HA signals were quantified more than three times in independent experiments. The phosphorylation level of Atg32-3HA in wild-type cells at the OD_{600} = 2.7 point was set to 100%. Data represent the averages of all experiments, with bars indicating standard deviations.

Supplementary Figure 3. (**a**) A model for Ppg1-Far-dependent suppression of Atg32 phosphorylation, Atg32-Atg11 interactions, and mitophagy. (**b**) Wild-type, *ppg1*Δ, *egd1*Δ, *ppg1*Δ *egd1*Δ, and *atg32*Δ cells expressing Atg32-3HA were grown in glycerol medium (Gly), collected at the indicated OD_{600} points, and subjected to western blotting. All strains are *atg7*-null derivatives, a protein essential for all autophagy-related processes, to avoid degradation of Atg32-3HA via mitophagy. Atg32-3HA signals normalized with Pgk1

(loading control) signals were quantified more than three times in independent experiments. The expression level of Atg32-3HA in wild-type cells at the OD_{600} = 1.2 point was set to 100%. Data represent the averages of all experiments, with bars indicating standard deviations. (**c**). Wild-type and *egd1Δ* cells (*atg7*-null derivatives) expressing full-length Atg32-3HA (*ATG32-3HA*), an Atg32 deletion mutants (Δ151–200) fused with 3HA (*(Δ151–200)-3HA*), or not expressing Atg32 were grown in glycerol medium (Gly), collected at the indicated OD_{600} points, and subjected to western blotting as (**b**). The expression level of Atg32-3HA in wild-type cells at the OD_{600} = 0.9 point was set to 100%. **(d)** Wild-type, *egd1Δ*, *egd1Δ ppg1Δ*, and *ppg1Δ* cells transformed with a plasmid encoding Atg32-3HA (p-*ATG32-3HA*), or an empty vector (EV) were grown in non-fermentable glycerol medium (Gly), collected at the indicated OD_{600} points, and subjected to western blotting. All strains are *atg15Δ atg32Δ* derivatives defective in intravacuolar degradation and mitophagy. Arrowheads indicate putative phosphorylated Atg32. Phosphorylated Atg32-3HA signals normalized with all Atg32-3HA signals were quantified more than three times in independent experiments. The phosphorylation level of Atg32-3HA in wild-type cells at the OD_{600} = 2.8 point was set to 100%. Data represent the averages of all experiments, with bars indicating standard deviations. *$P < 0.05$, **$P < 0.01$ (unpaired two-tailed Student's t-test). **(e)** Representative images of cells at the mid-log phase under respiratory conditions. Wild-type, *egd1Δ*, and *get3Δ* cells expressing Sec63-mCherry (as an ER marker) or mito-DHFR-mCherry (as a mitochondrial marker) and Far8-3×GFP (as a marker for the Ppg1-Far complex) were grown in glycerol medium (Gly), collected at the OD_{600} = 1.9-2.2 points, and observed under a fluorescence microscope. Scale bar, 5 μm. Cells (n= 100) with ER- or mitochondria-localized Far8-3×GFP signals were quantified in more than three experiments, and the average percentages were indicated on the bottom side of image panels.

Supplementary Table 1. Yeast strains used in this study

Name	Genotype
KOY76	BY4741 *his3Δ1 leu2Δ0 met15Δ0 ura3Δ0*
KOY136	BY4741 *pep4::kanMX6 prb1::hphNT1*
KOY184	BY4741 *pep4::kanMX6 prb1::hphNT1 atg7::natNT2*
KOY1387	BY4741 *his3::TEF*^{P}*-mito-DHFR-mCherry::CgHIS3*
KOY1422	BY4741 *his3::TEF*^{P}*-mito-DHFR-mCherry::CgHIS3 atg32::kanMX6*
KOY2075	BY4741 *sec63::SEC63-mCherry::KlURA3*
KOY2113	BY4741 *pot1::POT1-mCherry::CgHIS3*
KOY2326	BY4741 *atg32::ATG32-3HAn his3::TEF*^{P}*-mito-DHFR-mCherry::CgHIS3* [pRS315] [pRS316]
KOY2518	BY4741 *pot1::POT1-mCherry::CgHIS3 atg36::hphNT1*
KOY4943	BY4741*sec63::SEC63-mCherry::KlURA3 atg40::natNT2 atg39::kanMX6*
KOY5408	BY4741 *pep4::kanMX6 prb1::hphNT1 atg32::zeoNT3* [pRS316-*ATG32-3HAn*]
KOY5787	BY4741 *his3::TEF*^{P}*-mito-DHFR-mCherry::CgHIS3 vph1:: VPH1-GFP+::hphNT1*
KOY5789	BY4741 *his3::TEF*^{P}*-mito-DHFR-mCherry::CgHIS3 atg32::kanMX6 vph1::VPH1-GFP+::hphNT1*
KOY5836	BY4741 *tdh3::TDH3-mCherry::kanMX6*
KOY5870	BY4741 *tdh3::TDH3-mCherry::kanMX6 atg7::natNT2*
KOY5915	BY4741 *his3::TEF*^{P}*-mito-DHFR-mCherry::CgHIS3 atg32::ATG32-(3HA-3mGFP)n*
KOY6996	BY4741 *his3::TEF*^{P}*-mito-DHFR-mCherry::CgHIS3 atg32::ATG32-(3HA-3mGFP-3FLAG-LgBiT)n atg11::ATG11-HA-SmBiT::hphNT1*
KOY7812	BY4741 *his3::TEF*^{P}*-mito-DHFR-mCherry::CgHIS3 get3::natNT2 far8::FAR8-3xGFP::hphNT1*
KOY7821	BY4741 *sec63::SEC63-mCherry::KlURA3 far8::FAR8-3xGFP::hphNT1*
KOY7827	BY4741 *sec63::SEC63-mCherry::KlURA3 get3::natNT2 far8::FAR8-3xGFP::hphNT1*
KOY8007	BY4741 *his3::TEF*^{P}*-mito-DHFR-mCherry::CgHIS3 atg32::ATG32-(3HA-3mGFP-3FLAG-LgBiT)n atg11::ATG11-HA-SmBiT::hphNT1 egd1::natNT2*
KOY8077	BY4741 *his3::TEF*^{P}*-mito-DHFR-mCherry::CgHIS3 far8::FAR8-3xGFP::kanMX6*
KOY8152	BY4741 *tdh3::TDH3-mCherry::kanMX6 egd1::natNT2*
KOY8159	BY4741 *his3::TEF*^{P}*-mito-DHFR-mCherry::CgHIS3 atg32::ATG32-(3HA-3mGFP-3FLAG-LgBiT)n atg8::SmBiT-His8-3FLAG-ATG8::kanMX6*
KOY8854	BY4741 *atg32::ATG32-3HAn his3::TEF*^{P}*-mito-DHFR-mCherry::CgHIS3 ppg1::zeoNT3* [pRS315] [pRS316]
KOY9306	BY4741 *pot1::POT1-mCherry::CgHIS3 atg36::hphNT1* [pRS316-*ATG32(1-388)-pxTA-3HAn*]
KOY9318	BY4741 *pot1::POT1-mCherry::CgHIS3 atg36::hphNT1* [pRS316]
KOY9276	BY4741 *his3::TEF*^{P}*-mito-DHFR-mCherry::CgHIS3 atg32::kanMX6 atg7::hphNT1*
KOY9462	BY4741 *atg32::ATG32-3HAn his3::TEF*^{P}*-mito-DHFR-mCherry::CgHIS3 atg7::hphNT1 ppg1::zepNT3*
KOY9471	BY4741 *his3::TEF*^{P}*-mito-DHFR-mCherry::CgHIS3 atg32::ATG32-3HAn atg7::hphNT1*
KOY9474	BY4741 *his3::TEF*^{P}*-mito-DHFR-mCherry::CgHIS3 atg32::atg32(Δ151-200)-3HAn atg7::hphNT1*
KOY9549	BY4741 *his3::TEF*^{P}*-mito-DHFR-mCherry::CgHIS3 atg32::atg32(Δ151-200)-3HAn* [pRS315] [pRS316]
KOY10009	BY4741 *his3::TEF*^{P}*-mito-DHFR-mCherry::CgHIS3 egd1::natNT2*

KOY10010	BY4741 *his3::TEF^{P}-mito-DHFR-mCherry::CgHIS3 vph1::VPH1-GFP+::hphNT1 egd1::natNT2*
KOY10113	BY4741 *his3::TEF^{P}-mito-DHFR-mCherry::CgHIS3 egd2::natNT2*
KOY10114	BY4741 *his3::TEF^{P}-mito-DHFR-mCherry::CgHIS3 btt1::natNT2*
KOY10115	BY4741 *sec63::SEC63-mCherry::KlURA3 egd1::natNT2*
KOY10119	BY4741 *pot1::POT1-mCherry::CgHIS3 egd2::natNT2*
KOY10130	BY4741 *his3::TEF^{P}-mito-DHFR-mCherry::CgHIS3 vph1::VPH1-GFP+::hphNT1 egd2::natNT2*
KOY10143	BY4741 *his3::TEF^{P}-mito-DHFR-mCherry::CgHIS3 atg32::kanMX6* [pRS315] [pRS316]
KOY10158	BY4741 *sec63::SEC63-mCherry::KlURA3 egd2::natNT2*
KOY10277	BY4741 *pot1::POT1-mCherry::CgHIS3 egd2::natNT2*
KOY10317	BY4741 *his3::TEF^{P}-mito-DHFR-mCherry::CgHIS3 vph1::VPH1-GFP+::hphNT1 btt1::natNT2*
KOY10401	BY4741 *tdh3::TDH3-mCherry::kanMX6 egd2::zeoNT3*
KOY10402	BY4741 *tdh3::TDH3-mCherry::kanMX6 egd1::natNT2 egd2::zeoNT3*
KOY10407	BY4741 *his3::TEF^{P}-mito-DHFR-mCherry::CgHIS3 egd1::natNT2 egd2::zeoNT3*
KOY10409	BY4741 *pot1::POT1-mCherry::CgHIS3 egd1::natNT2 egd2::zeoNT3*
KOY10413	BY4741 *sec63::SEC63-mCherry::KlURA3 egd1::natNT2 egd2::zeoNT3*
KOY10416	BY4741 *his3::TEF^{P}-mito-DHFR-mCherry::CgHIS3 vph1::VPH1-GFP+::hphNT1 egd1::natNT2 egd2::zeoNT3*
KOY10699	BY4741 *his3::TEF^{P}-mito-DHFR-mCherry::CgHIS3 atg32::ATG32-(3HA-3mGFP-3FLAG-LgBiT)n atg8::SmBiT-His8-3FLAG-ATG8::kanMX6 egd1::natNT2*
KOY10806	BY4741 *pep4::kanMX6 prb1::hphNT1 atg32::zeoNT3 egd1::natNT2* [pRS316-*ATG32-3HAn*]
KOY10897	BY4741 *his3::TEF^{P}-mito-DHFR-mCherry::CgHIS3 btt1::natNT2 egd1::kanMX6*
KOY10909	BY4741 *atg15::natNT2 atg32::kanMX6* [pRS316-*ATG32-3HAn*]
KOY10961	BY4741 *atg32::ATG32-3HAn his3::TEF^{P}-mito-DHFR-mCherry::CgHIS3 atg7::hphNT1 ppg1::zeoNT3 egd1::natNT2*
KOY10964	BY4741 *his3::TEF^{P}-mito-DHFR-mCherry::CgHIS3 far8::FAR8-3xGFP::kanMX6 egd1::natNT2*
KOY10973	BY4741 *his3::TEF^{P}-mito-DHFR-mCherry::CgHIS3 atg32::ATG32-3HAn atg7::hphNT1 egd1::kanMX6*
KOY11010	BY4741 *his3::TEF^{P}-mito-DHFR-mCherry::CgHIS3 atg32::KlURA3 atg32::atg32(Δ151-200)-3HA egd1::natNT2* [pRS315] [pRS316]
KOY11011	BY4741 *atg32::ATG32-3HAn his3::TEF^{P}-mito-DHFR-mCherry::CgHIS3 egd1::hphNT1* [pRS315] [pRS316]
KOY11018	BY4741 *atg32::ATG32-3HAn his3::TEF^{P}-mito-DHFR-mCherry::CgHIS3 ppg1::zeoNT3 egd1::natNT2* [pRS315] [pRS316]
KOY11023	BY4741 *sec63::SEC63-mCherry::KlURA3 far8::FAR8-3xGFP::hphNT1 egd1::kanMX6*
KOY11166	BY4741 *his3::TEF^{P}-mito-DHFR-mCherry::CgHIS3 atg32::atg32(Δ151-200)-3HA egd1::natNT2 atg7::zeoNT3*
KOY11697	BY4741 *pep4::kanMX6 prb1::hphNT1 atg32::zeoNT3* [pRS316]
KOY11984	BY4741 *pep4::kanMX6 prb1::hphNT1 egd1::natNT2*
KOY12003	BY4741 *his3::TEF^{P}-mito-DHFR-mCherry::CgHIS3 vph1::VPH1-GFP+::hphNT1 egd1::natNT2 btt1::zeoNT3*
KOY12012	BY4741 *atg15::natNT2 atg32::kanMX6 egd1::hphNT1* [pRS316-*ATG32-3HAn*]
KOY12033	BY4741 *atg15::natNT2 atg32::kanMX6 egd1::hphNT1 ppg1::zeoNT3* [pRS316-*ATG32-3HAn*]

KOY12036	BY4741 *atg15::natNT2 atg32::kanMX6 ppg1::hphNT1* [pRS316-*ATG32-3HAn*]
KOY12061	BY4741 *pep4::kanMX6 prb1::hphNT1 atg36::zeoNT3* [pRS316]
KOY12075	BY4741 *pep4::kanMX6 prb1::hphNT1 atg36::zeoNT3* [pRS316-*ATG32(1-388)-pxTA-3HAn*]
KOY12078	BY4741 *pep4::kanMX6 prb1::hphNT1 egd1::natNT2 atg36::zeoNT3* [pRS316-*ATG32(1-388)-pxTA-3HAn*]
KOY12213	BY4741 *pot1::POT1-mCherry::CgHIS3 atg36::hphNT1 egd1::natNT2* [pRS316-*ATG32(1-388)-pxTA-3HAn*]
KOY12225	BY4741 *pep4::kanMX6 prb1::hphNT1 atg11::natNT2*
KOY12228	BY4741 *pep4::kanMX6 prb1::hphNT1 atg8::natNT2*
KOY12334	BY4741 *atg15::natNT2 atg32::kanMX6* [pRS316]
KOY12337	BY4741 *pep4::kanMX6 prb1::hphNT1 atg32::zeoNT3 egd2::natNT2* [pRS316-*ATG32-3HAn*]
KOY12340	BY4741 *pep4::kanMX6 prb1::hphNT1 atg32::zeoNT3 btt1::natNT2* [pRS316-*ATG32-3HAn*]

Supplementary Table 2. Plasmid used in this study

**Name**	**Relevant characteristics**
pRS315	*CEN LEU2*
pRS316	*CEN URA3*
pRS315-*ATG32-3HA*	*CEN LEU2 580-bp 5'-UTR and 744-bp 3'-UTR from ATG32*
pRS316-*ATG32-3HA*	*CEN URA3 580-bp 5'-UTR and 744-bp 3'-UTR from ATG32*
pRS316-*ATG32(1-388)-pxTA-3HA*	*CEN URA3 580-bp 5'-UTR and 744-bp 3'-UTR from ATG32 atg32 (1-388)+Pex15 (315-383)+3HA*

a

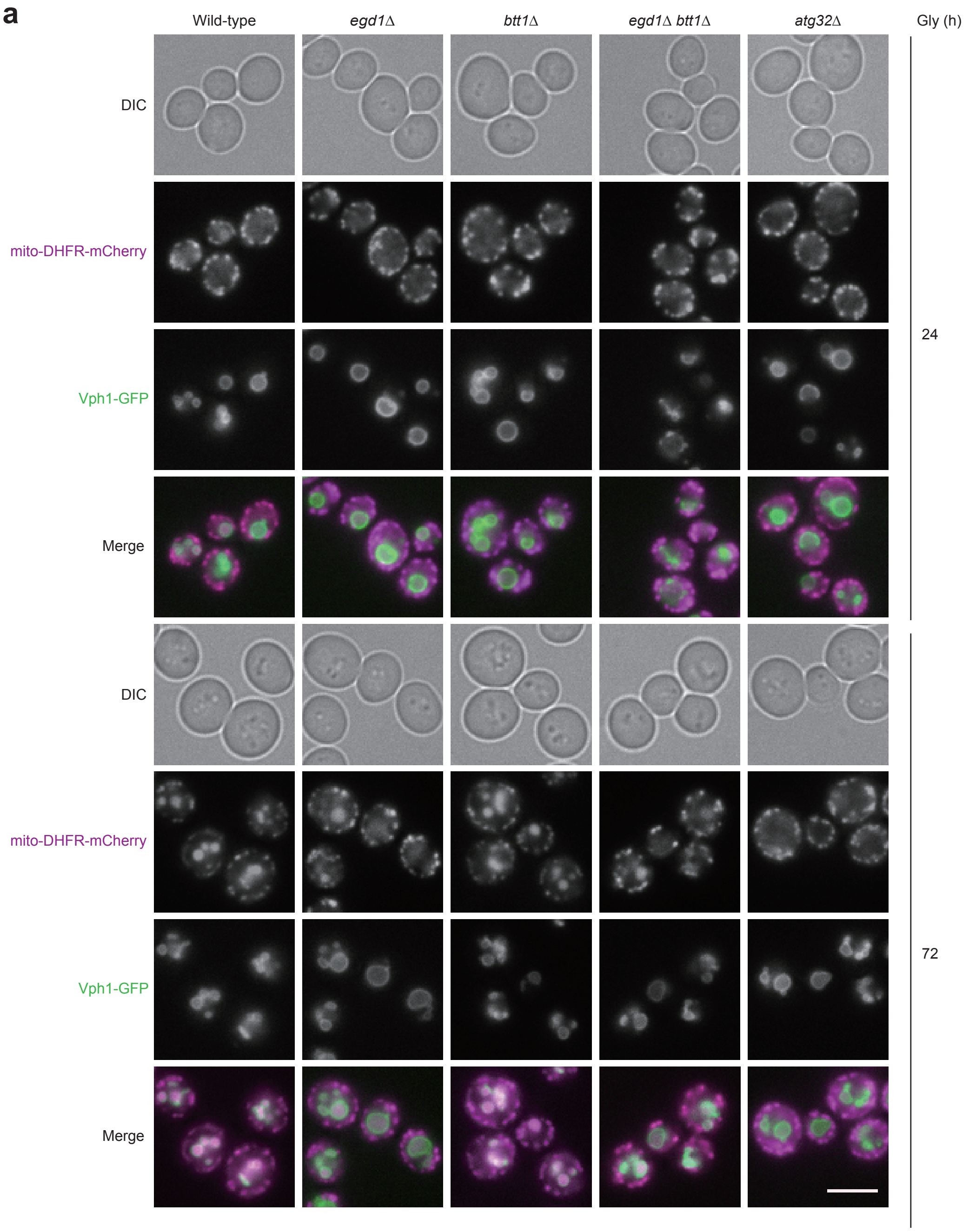
Wild-type
egd1Δ
btt1Δ
egd1Δ btt1Δ
atg32Δ
Gly (h)
DIC
mito-DHFR-mCherry
Vph1-GFP
Merge
24
DIC
mito-DHFR-mCherry
Vph1-GFP
Merge
72

b

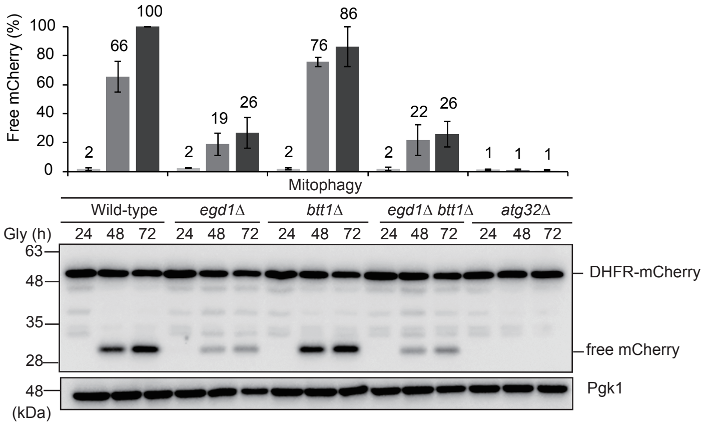
Free mCherry (%)
100
80
60
40
20
0
2
66
100
2
19
26
2
76
86
2
22
26
1
1
1
Mitophagy
Wild-type
egd1Δ
btt1Δ
egd1Δ btt1Δ
atg32Δ
Gly (h)
24 48 72 24 48 72 24 48 72 24 48 72 24 48 72
63
48
35
28
48
(kDa)
DHFR-mCherry
free mCherry
Pgk1

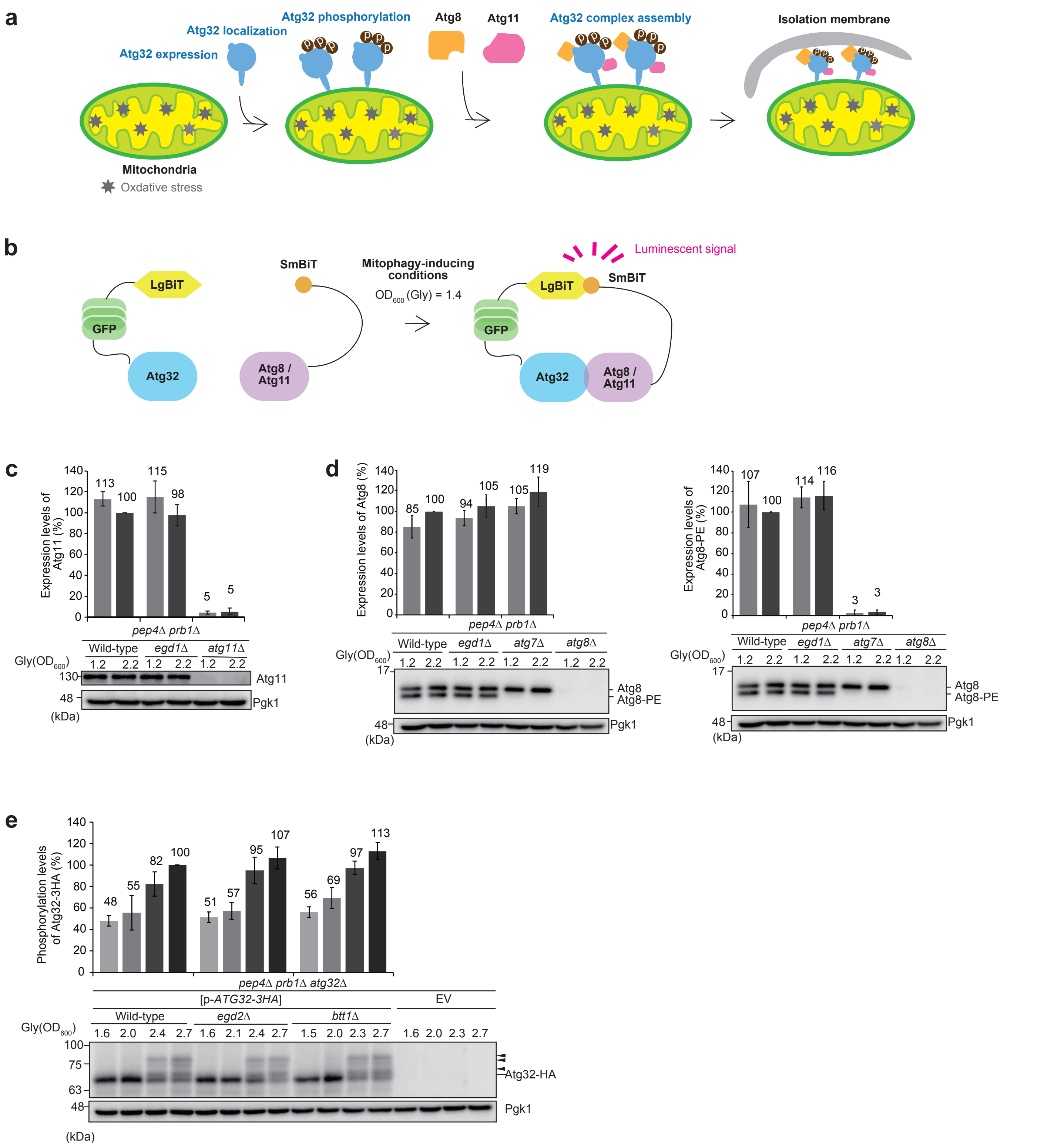
a
Atg32 expression
Atg32 localization
Atg32 phosphorylation
Atg8
Atg11
Atg32 complex assembly
Isolation membrane
Mitochondria
Oxdative stress
b
LgBiT
GFP
Atg32
SmBiT
Atg8 / Atg11
Mitophagy-inducing conditions
OD600 (Gly) = 1.4
Luminescent signal
c
Expression levels of Atg11 (%)
pep4Δ prb1Δ
Wild-type
egd1Δ
atg11Δ
Gly(OD600)
Atg11
Pgk1
(kDa)
d
Expression levels of Atg8 (%)
Expression levels of Atg8-PE (%)
atg7Δ
atg8Δ
Atg8
Atg8-PE
e
Phosphorylation levels of Atg32-3HA (%)
pep4Δ prb1Δ atg32Δ
[p-ATG32-3HA]
EV
egd2Δ
btt1Δ
Atg32-HA

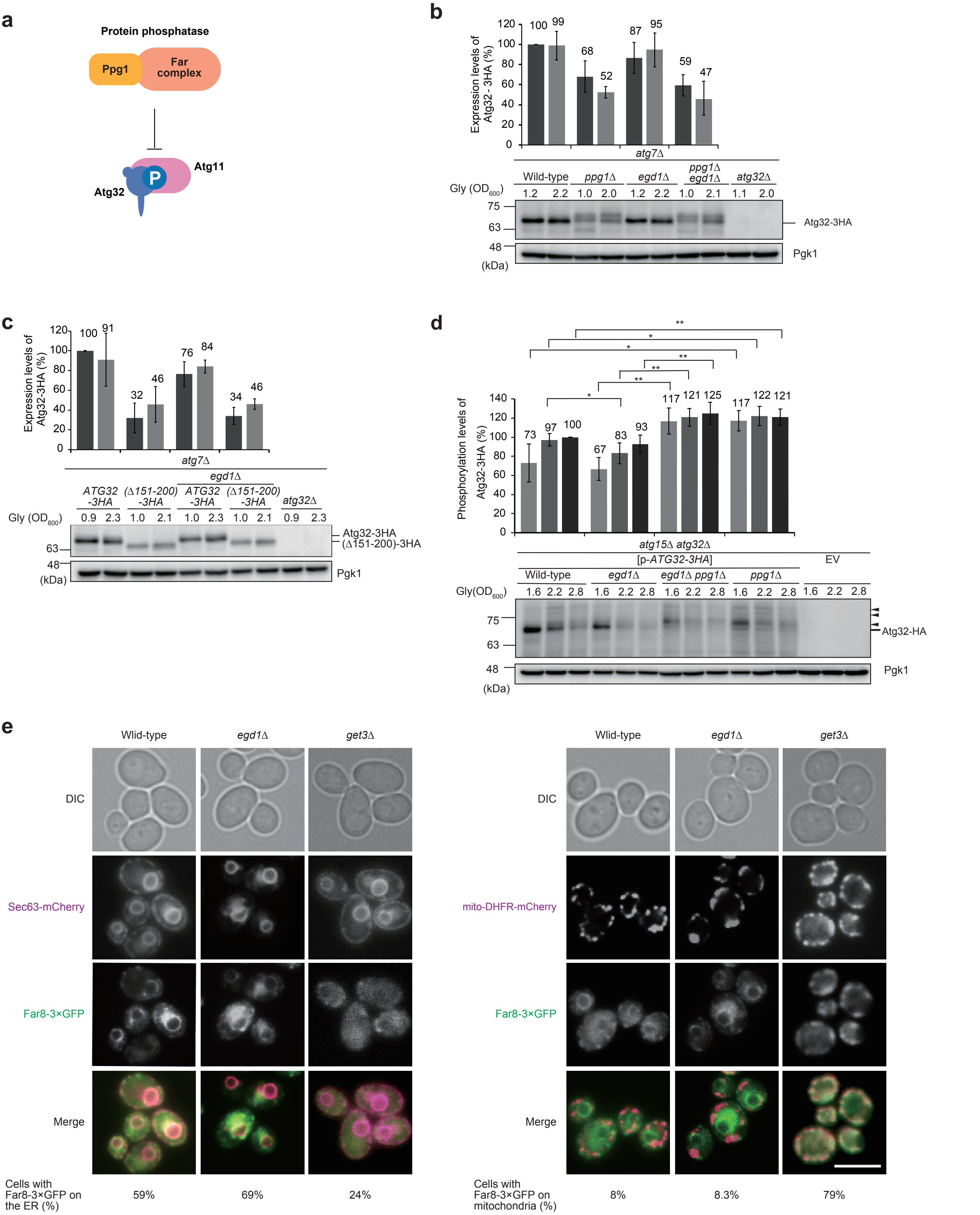
a
Protein phosphatase
Ppg1
Far complex
Atg32
P
Atg11
b
Expression levels of Atg32 - 3HA (%)
100 99 68 52 87 95 59 47
atg7Δ
Wild-type ppg1Δ egd1Δ ppg1Δ egd1Δ atg32Δ
Gly (OD600)
1.2 2.2 1.0 2.0 1.2 2.2 1.0 2.1 1.1 2.0
Atg32-3HA
Pgk1
(kDa)
c
Expression levels of Atg32-3HA (%)
100 91 32 46 76 84 34 46
atg7Δ
egd1Δ
ATG32 -3HA
(Δ151-200) -3HA
atg32Δ
Gly (OD600)
0.9 2.3 1.0 2.1 1.0 2.3 1.0 2.1 0.9 2.3
Atg32-3HA
(Δ151-200)-3HA
Pgk1
(kDa)
d
Phosphorylation levels of Atg32-3HA (%)
73 97 100 67 83 93 117 121 125 117 122 121
atg15Δ atg32Δ
[p-ATG32-3HA]
Wild-type egd1Δ egd1Δ ppg1Δ ppg1Δ EV
Gly(OD600)
1.6 2.2 2.8 1.6 2.2 2.8 1.6 2.2 2.8 1.6 2.2 2.8 1.6 2.2 2.8
Atg32-HA
Pgk1
(kDa)
e
Wlid-type egd1Δ get3Δ
DIC
Sec63-mCherry
Far8-3×GFP
Merge
Cells with Far8-3×GFP on the ER (%)
59% 69% 24%
Wlid-type egd1Δ get3Δ
DIC
mito-DHFR-mCherry
Far8-3×GFP
Merge
Cells with Far8-3×GFP on mitochondria (%)
8% 8.3% 79%

Notice: Although all the original Pgk1 blots and HA blot in Fig. 3a and mCherry blots in Fig. 3g, 4b, and S1b have their blot edges only slightly visible, the attached original PVDF membranes clearly certifies that all the original blots have been neither cropped nor processed.

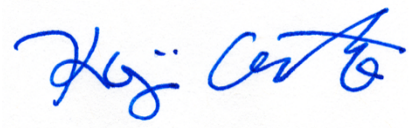

Koji Okamoto, Ph.D.
Corresponding author

Fig. 1b

Original PVDF membrane

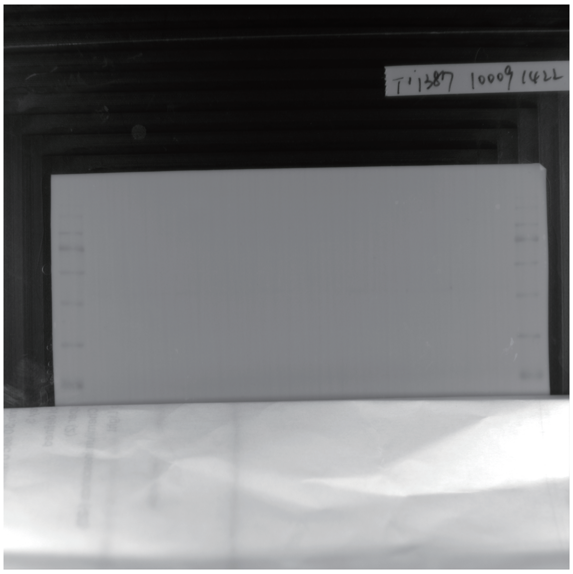

ECL signal-detected blot

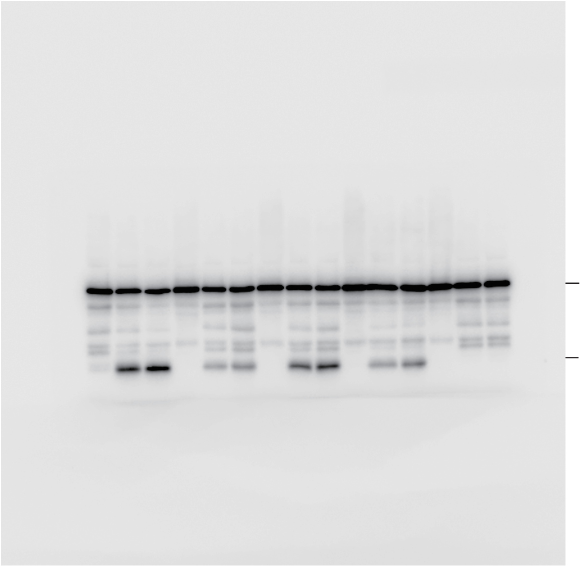

DHFR-mCherry

free mCherry

Original PVDF membrane

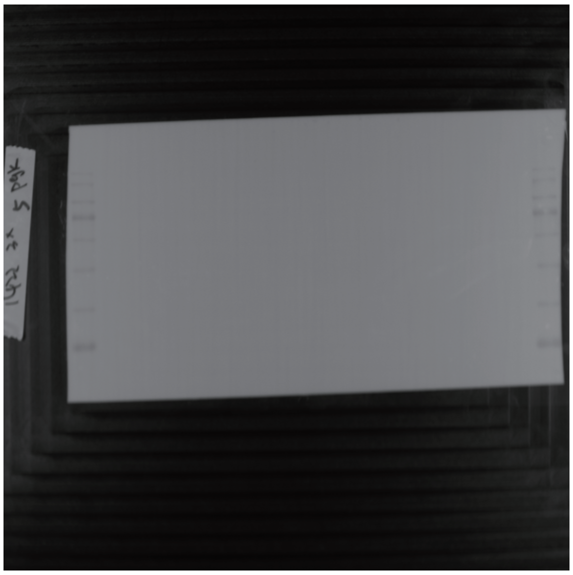

ECL signal-detected blot

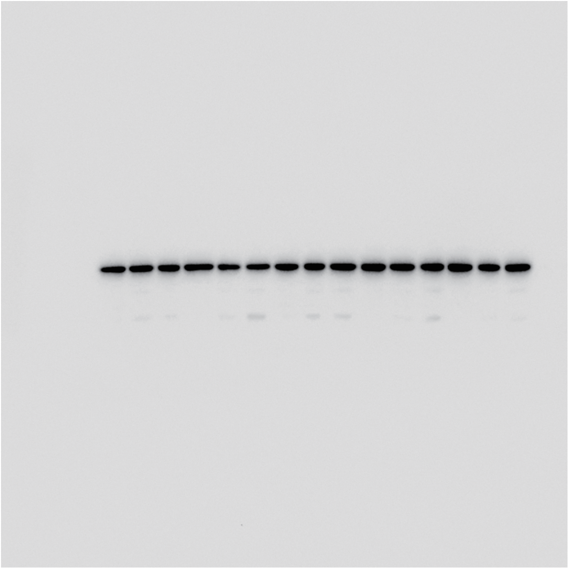

Pgk1

Fig. 2a

Original PVDF membrane

ECL signal-detected blot

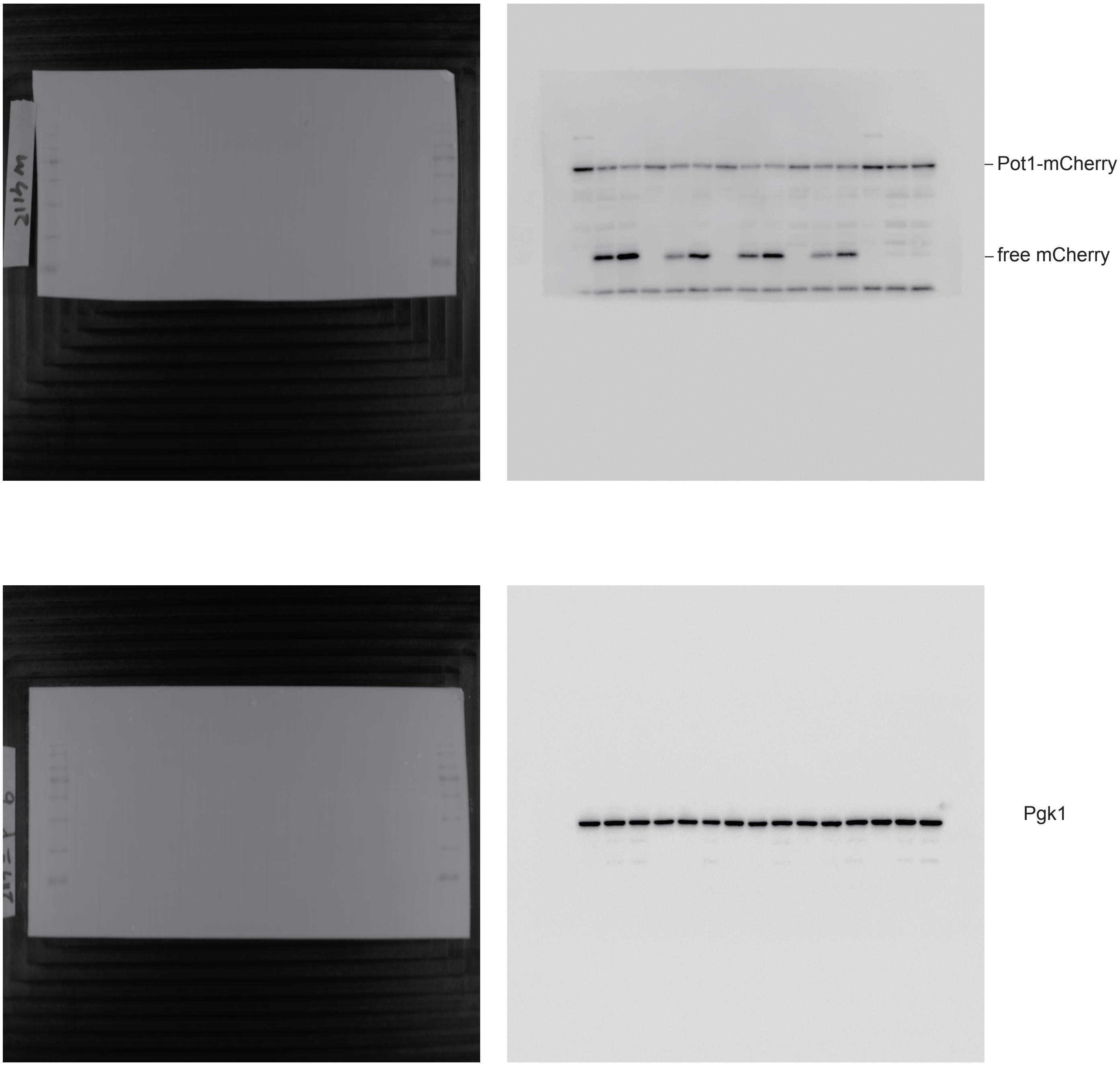

Fig. 2b

Original PVDF membrane

ECL signal-detected blot

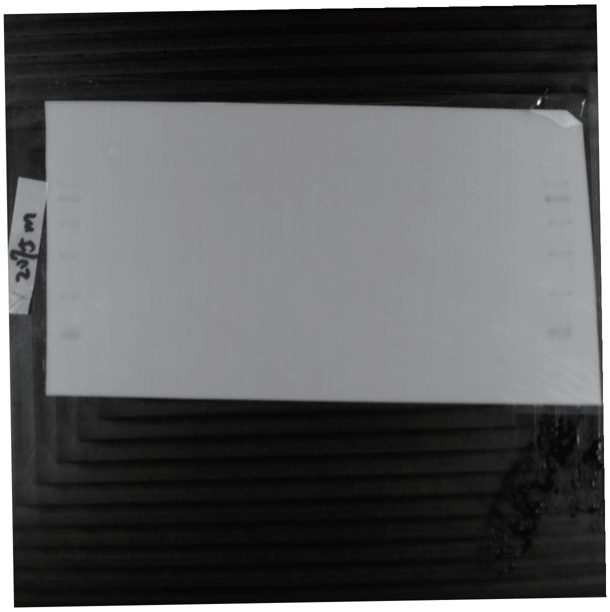

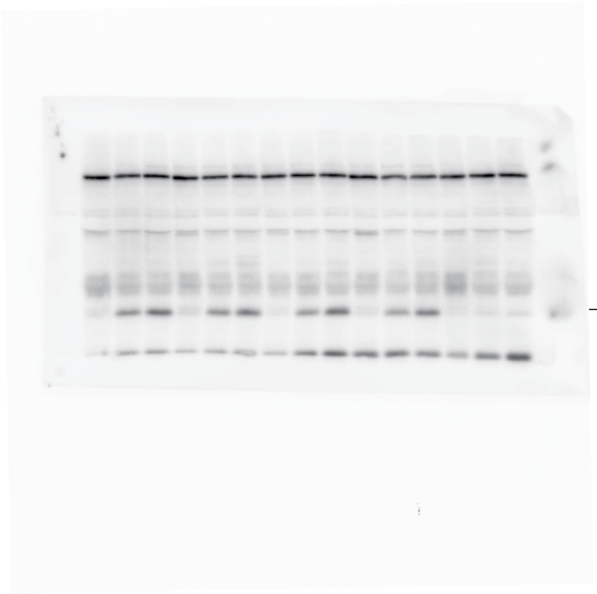

– Sec63-mCherry

–free mCherry

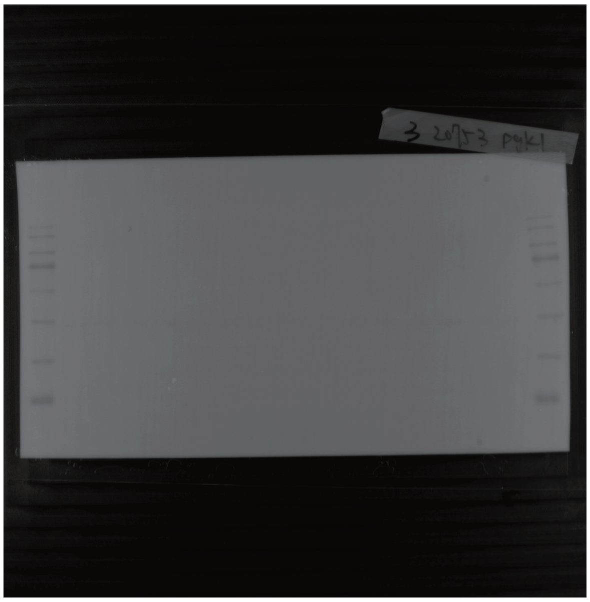

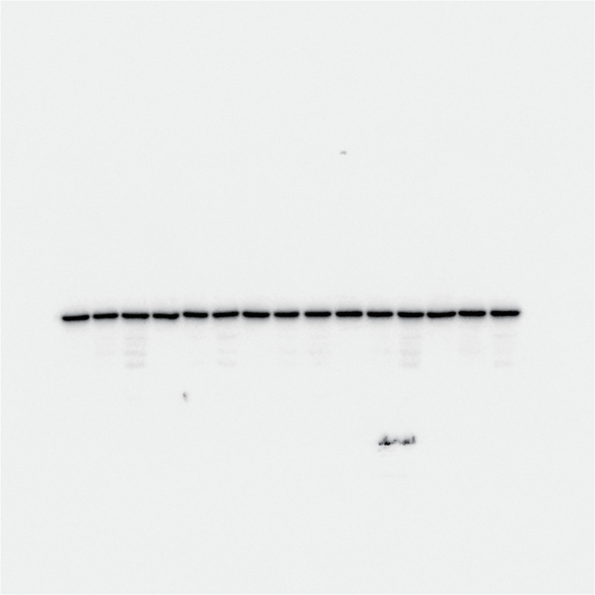

Pgk1

Fig. 2c

Original PVDF membrane

ECL signal-detected blot

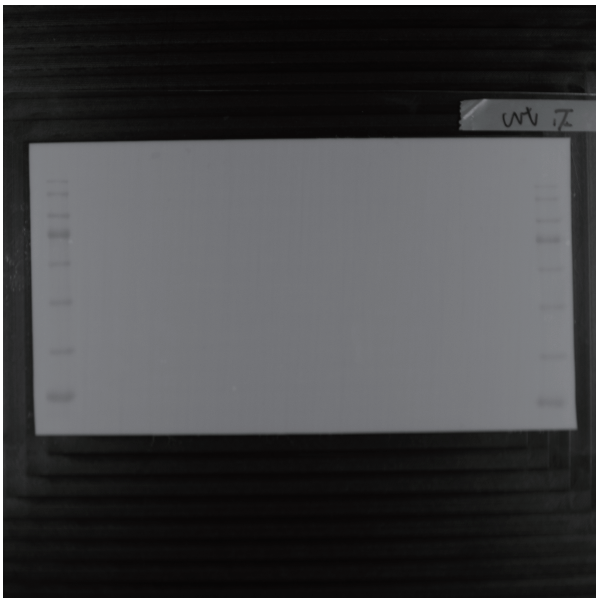

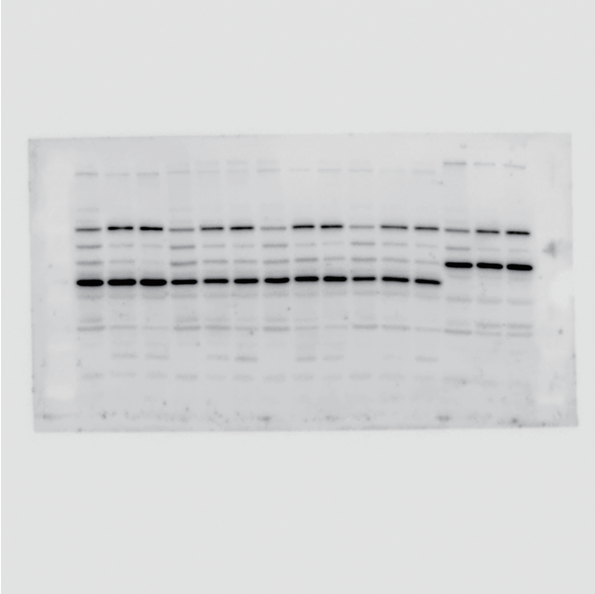

Ape1 precursor form
Ape1 mature form

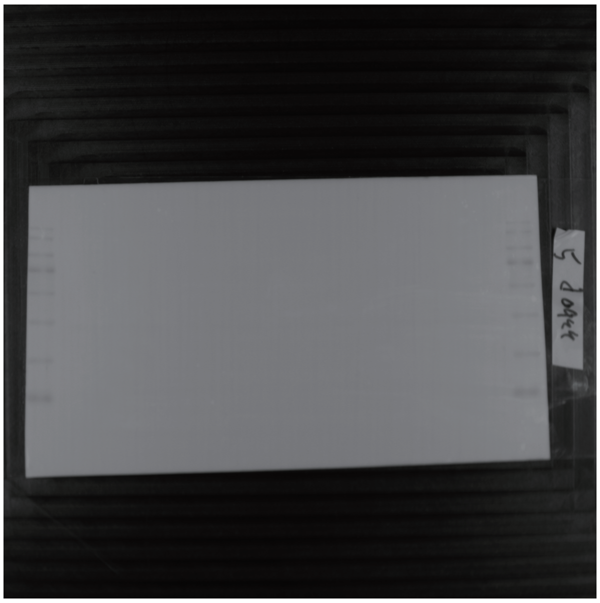

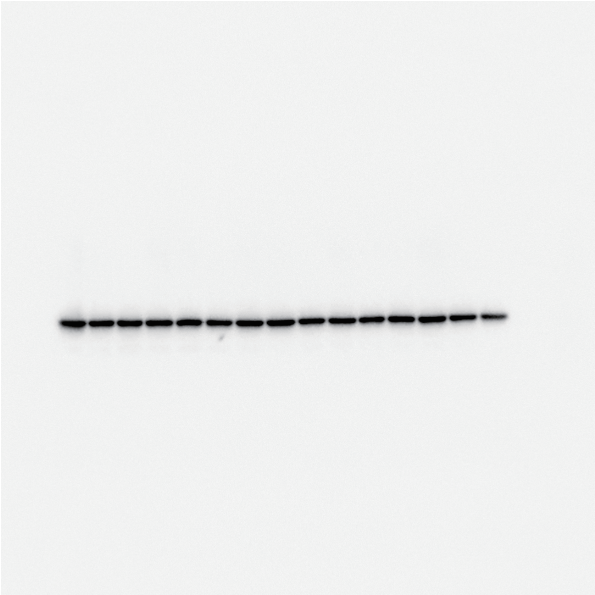

Pgk1

Fig. 2d

Original PVDF membrane

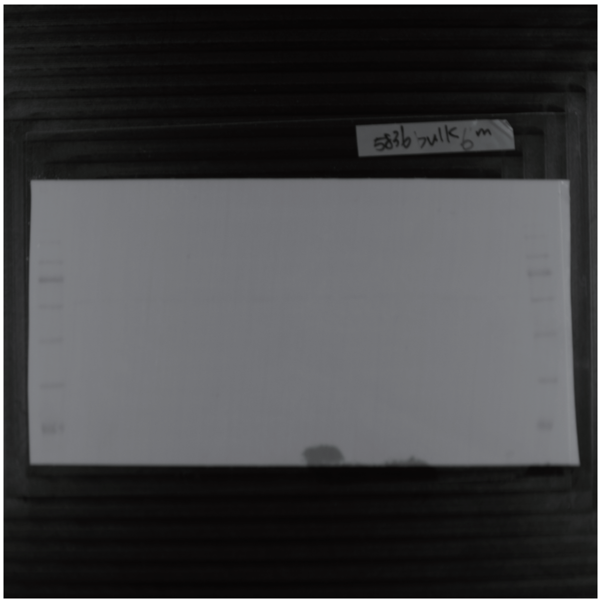

ECL signal-detected blot

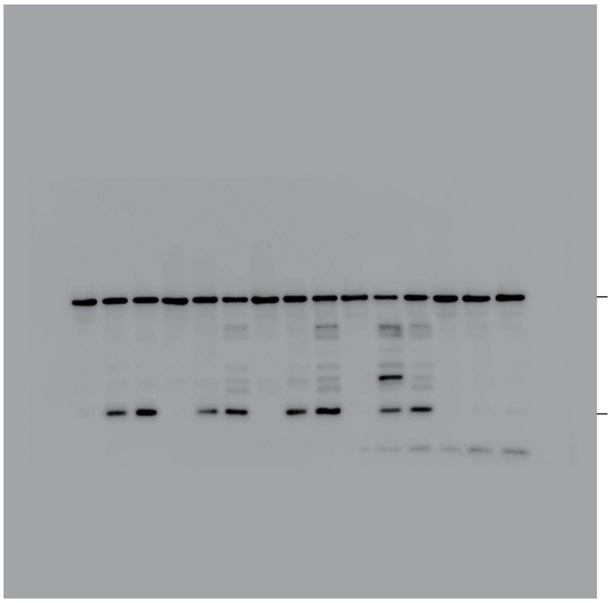

Tdh3-mCherry

free mCherry

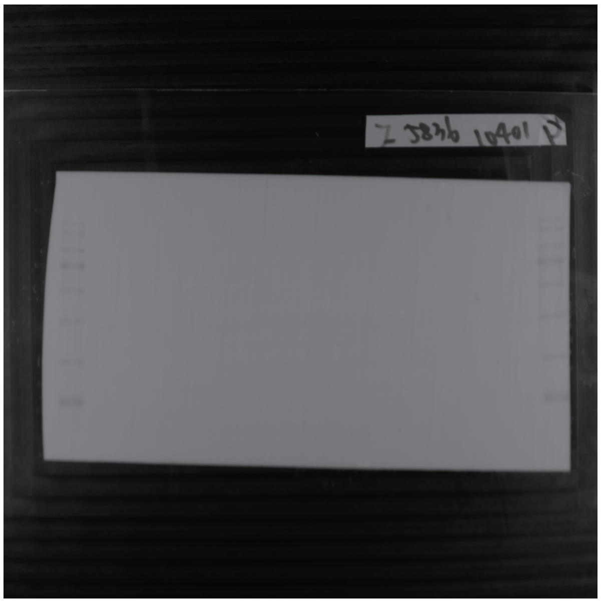

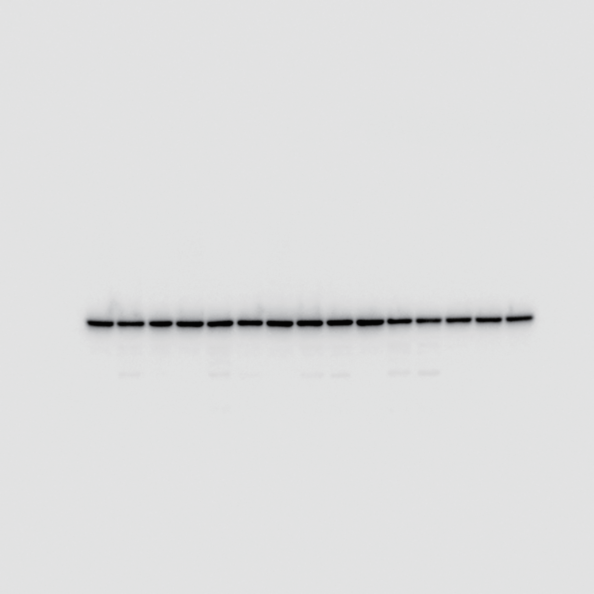

Pgk1

Fig. 3a

Original PVDF membrane ECL signal-detected blot

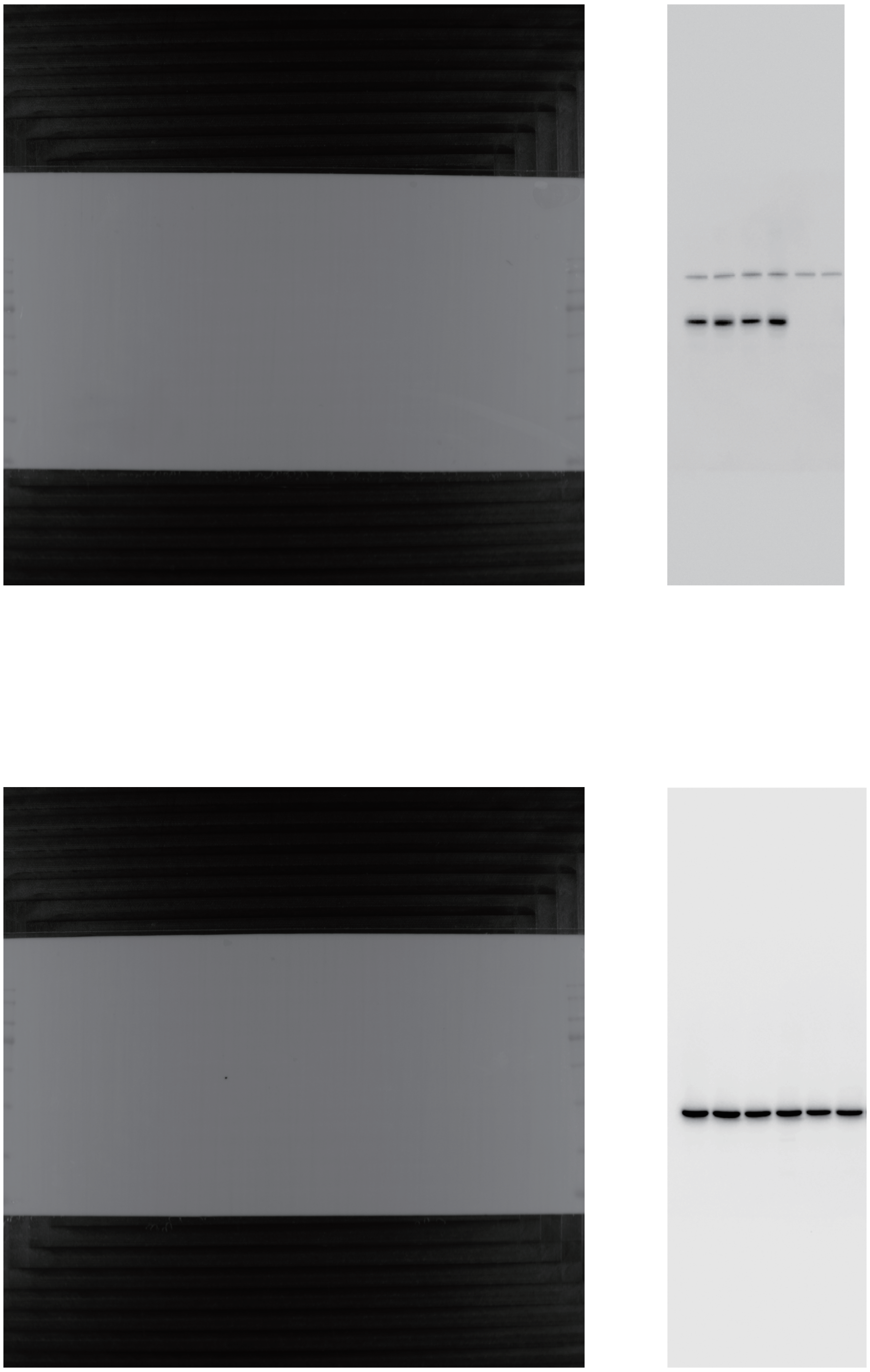

Atg32-HA

Pgk1

Original PVDF membrane

ECL signal-detected blot

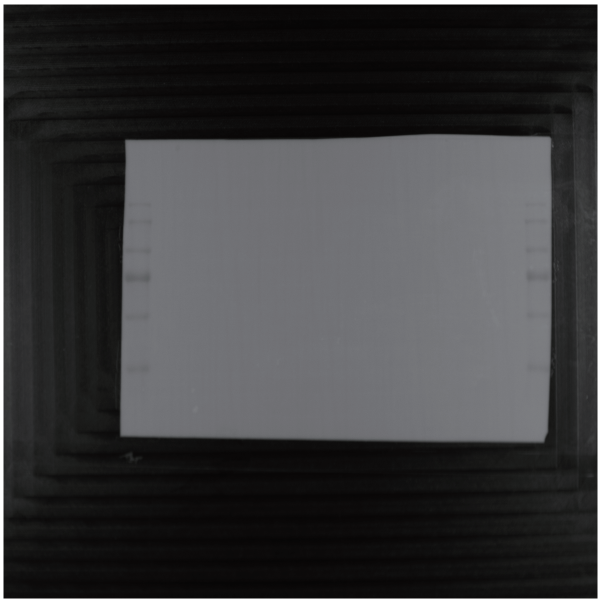

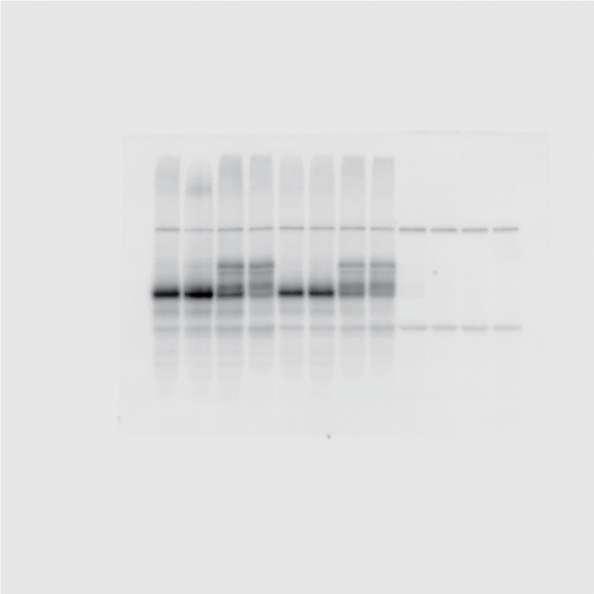

Atg32-HA

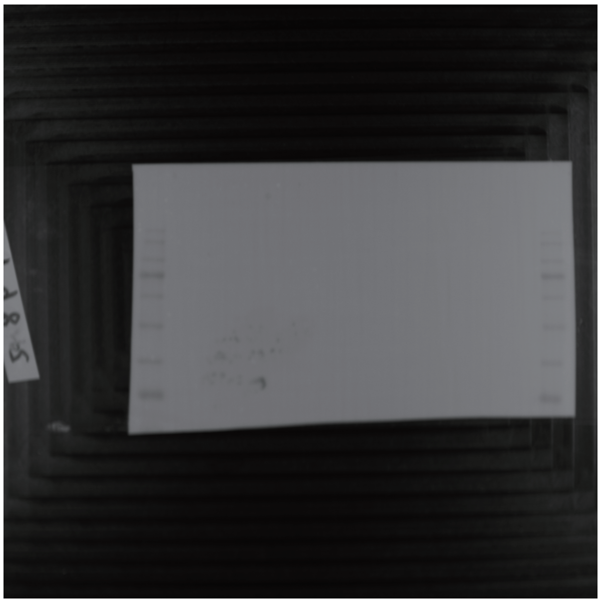

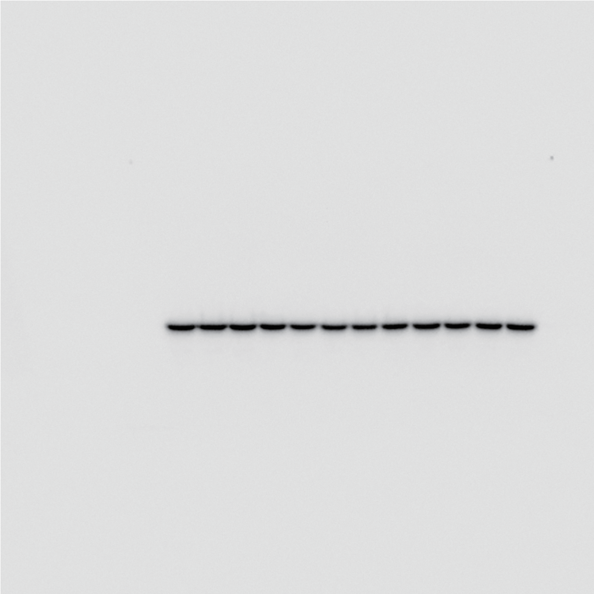

Pgk1

Fig. 3f

Original PVDF membrane

ECL signal-detected blot

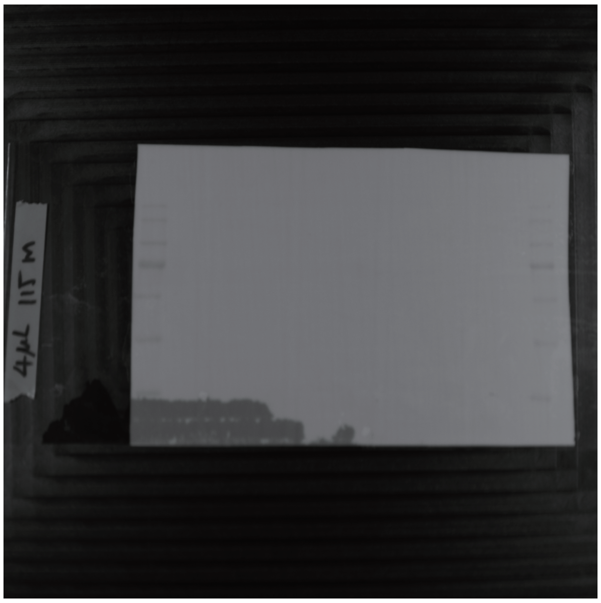

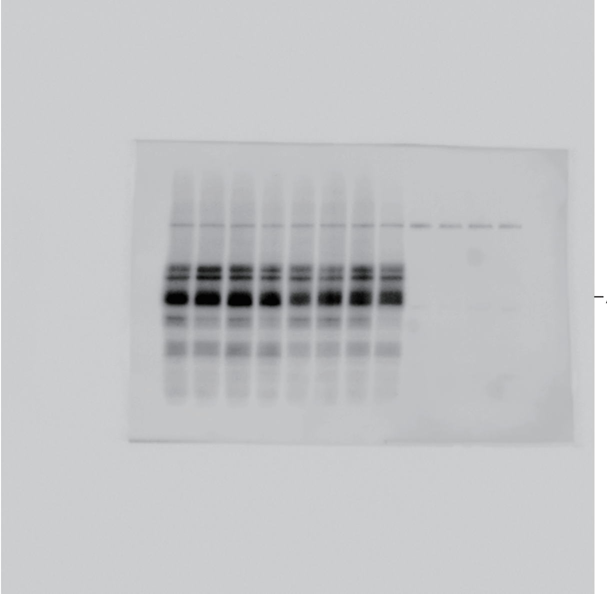

Atg32(1-388)-pxTA-3HA

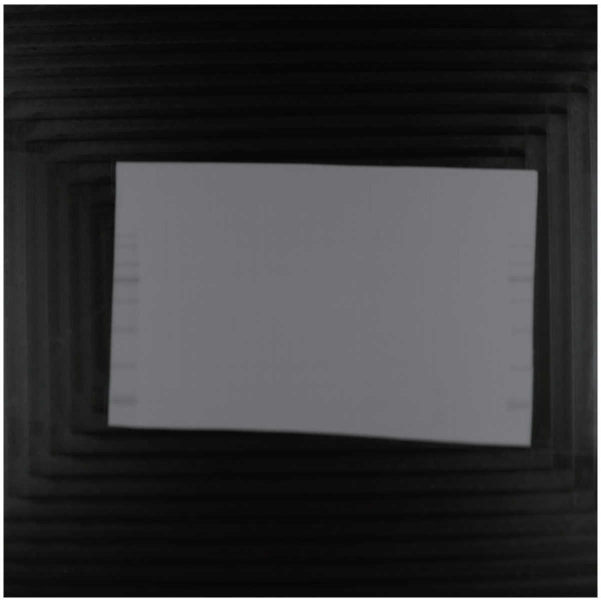

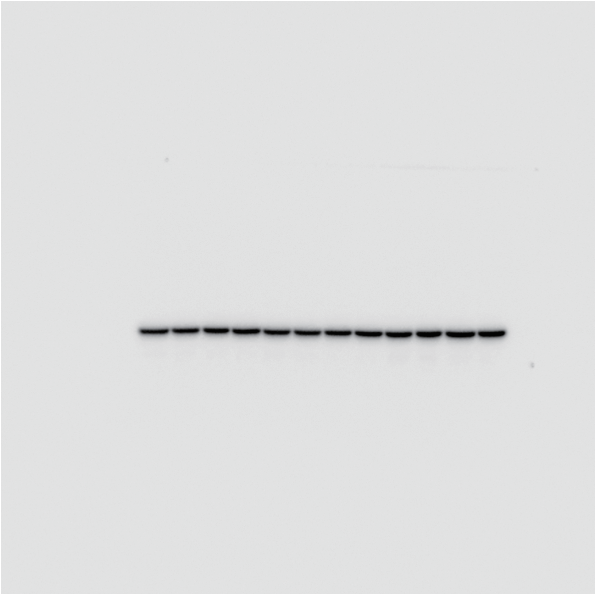

Pgk1

Fig. 3g

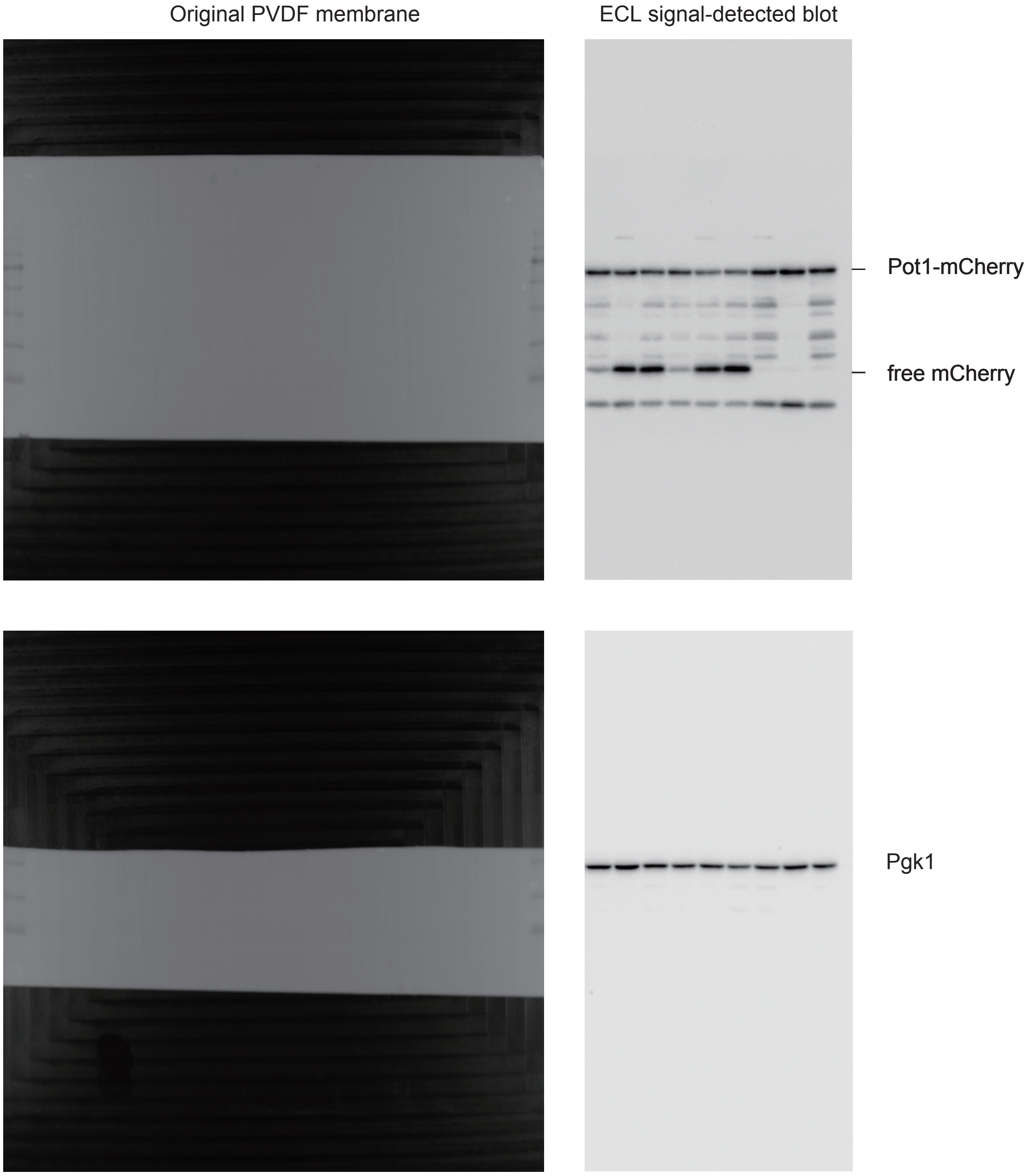
Original PVDF membrane
ECL signal-detected blot
Pot1-mCherry
free mCherry
Pgk1

Fig. 4a

Original PVDF membrane

ECL signal-detected blot

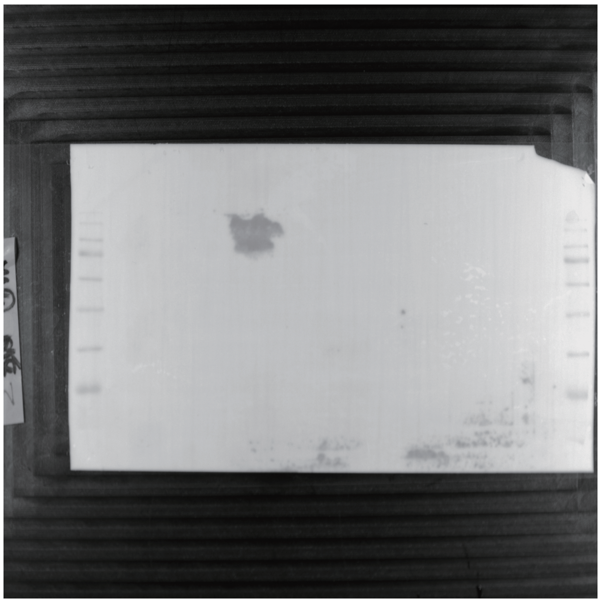

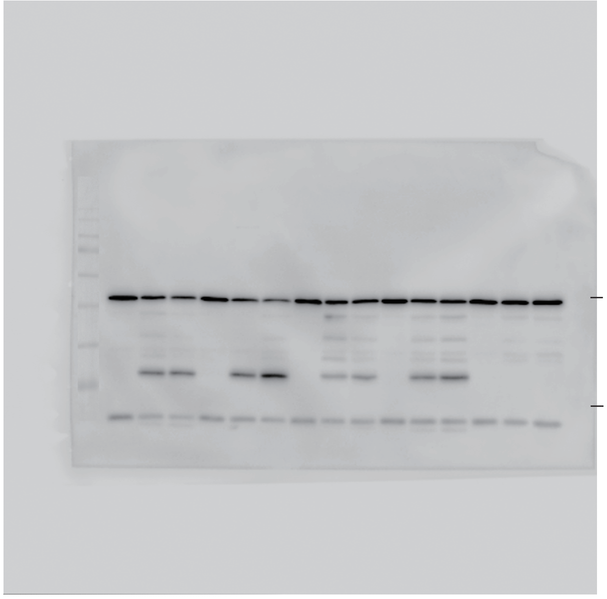

DHFR-mCherry

free mCherry

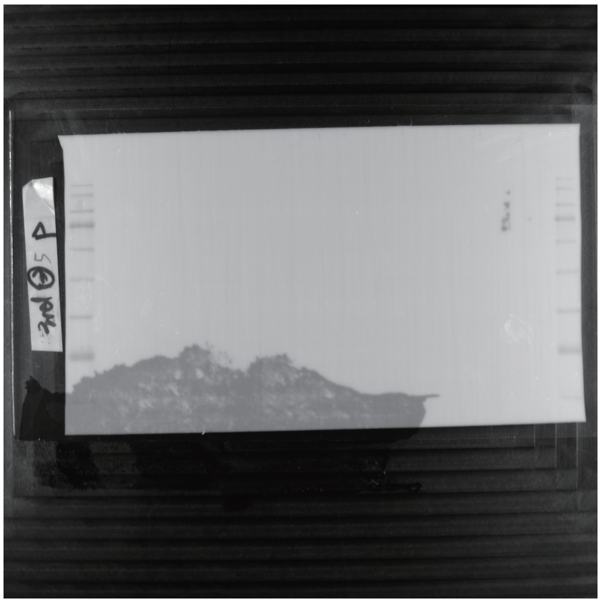

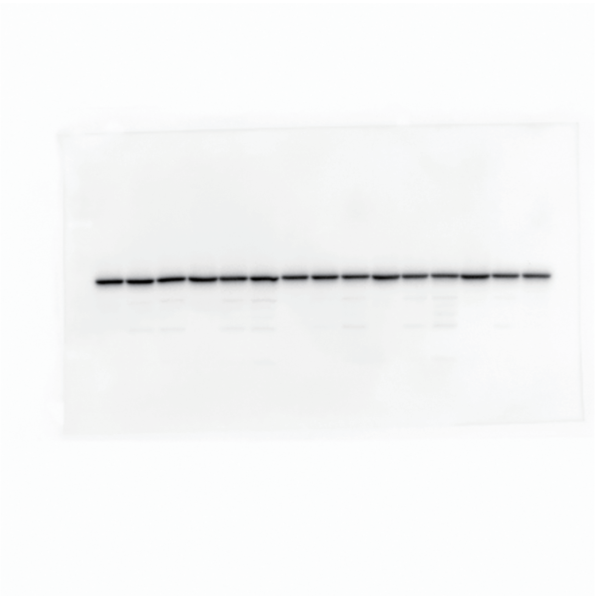

Pgk1

Fig. 4b

Original PVDF membrane

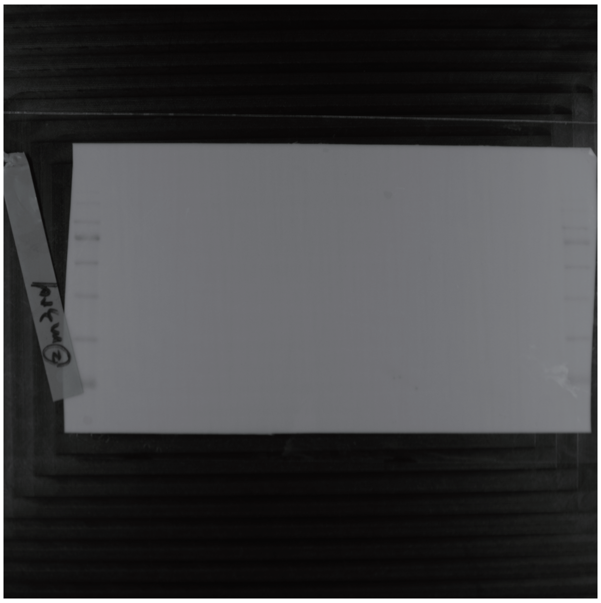

ECL signal-detected blot

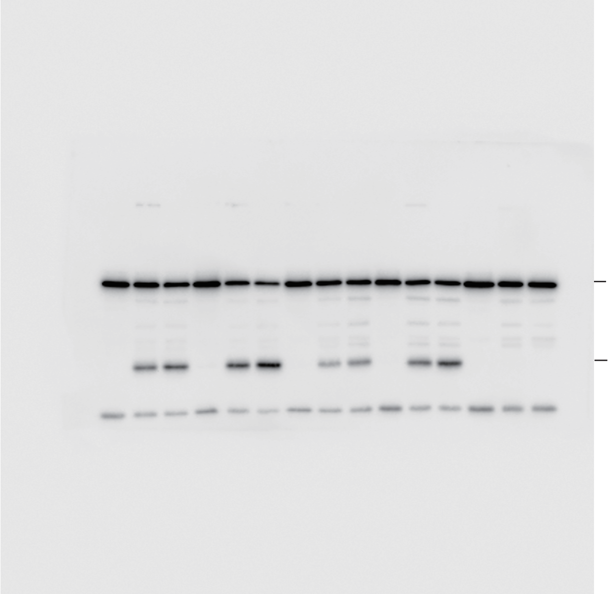

– DHFR-mCherry

– free mCherry

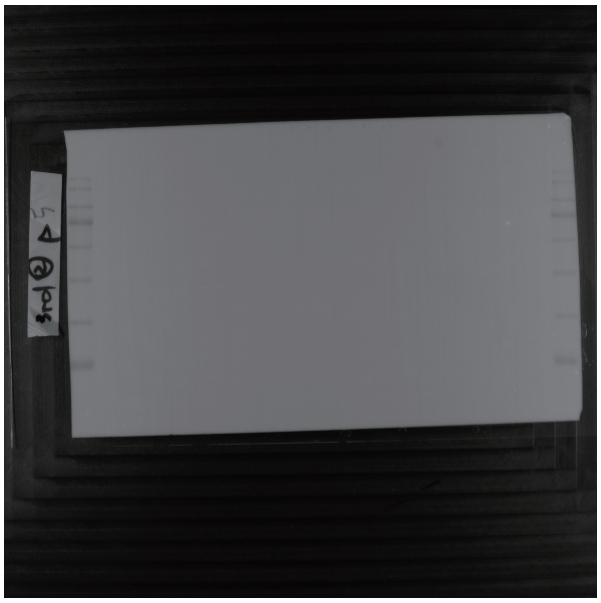

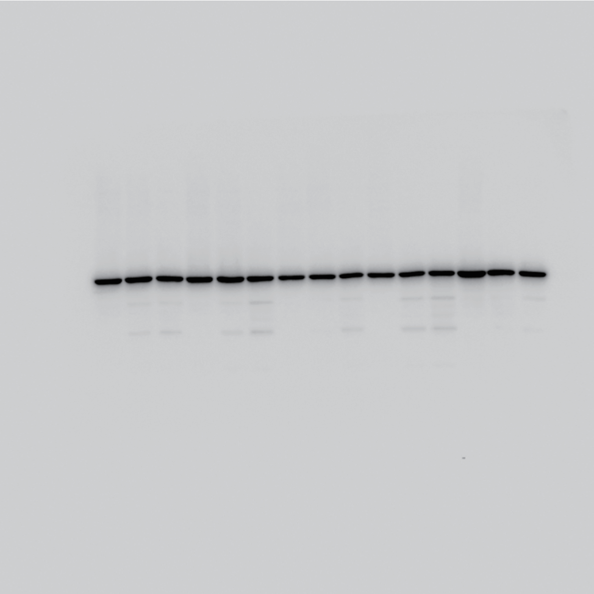

Pgk1

Fig. S1b

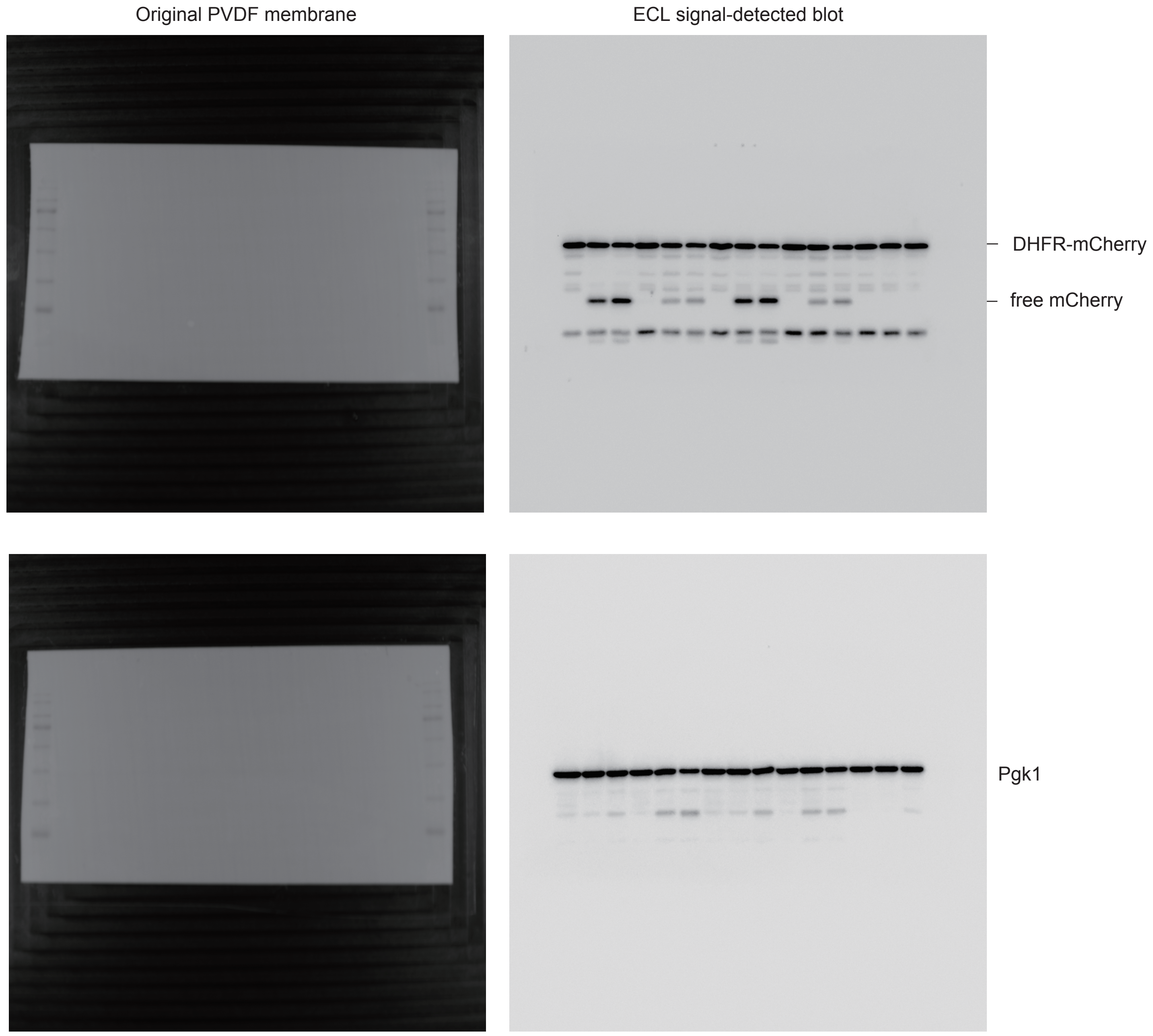
Original PVDF membrane
ECL signal-detected blot
DHFR-mCherry
free mCherry
Pgk1

Fig. S2c

Original PVDF membrane

ECL signal-detected blot

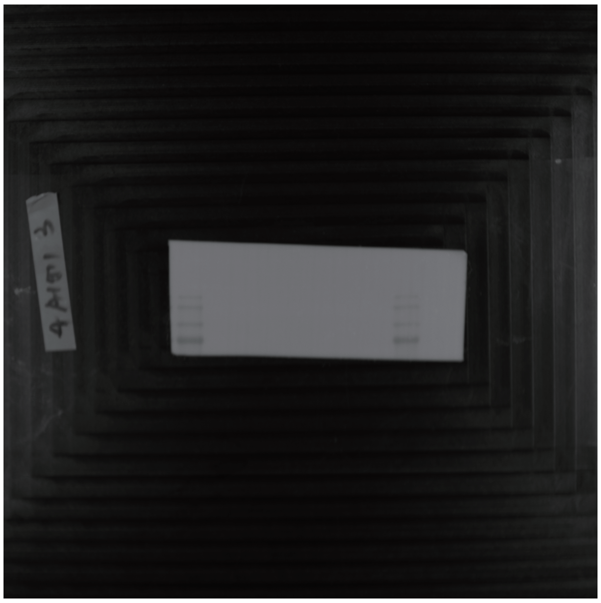

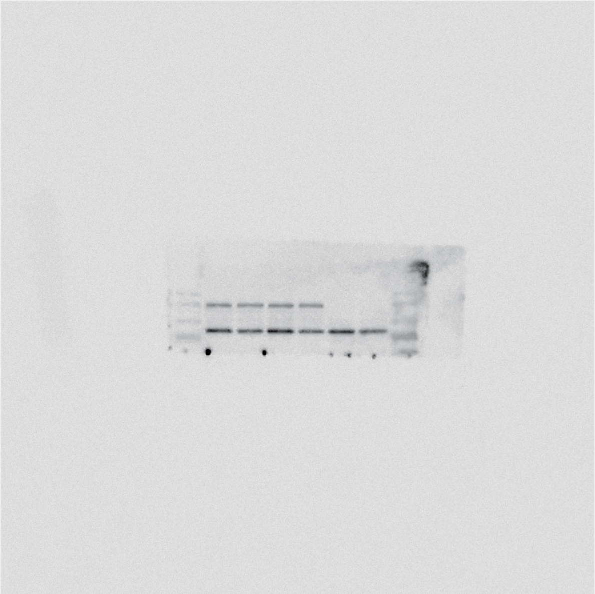

Atg11

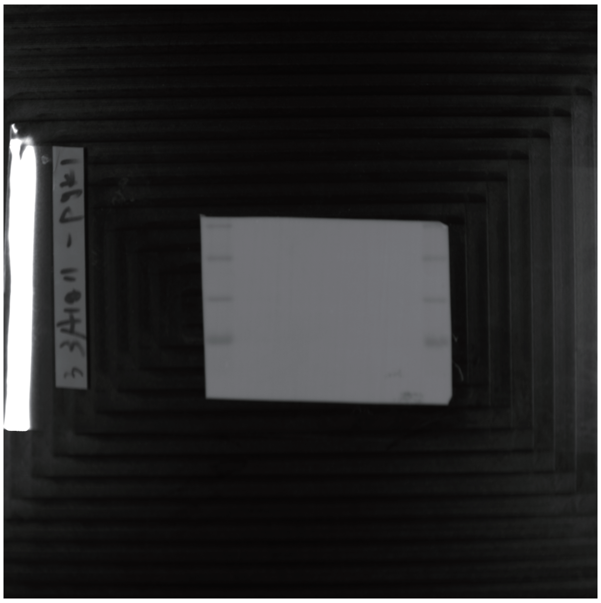

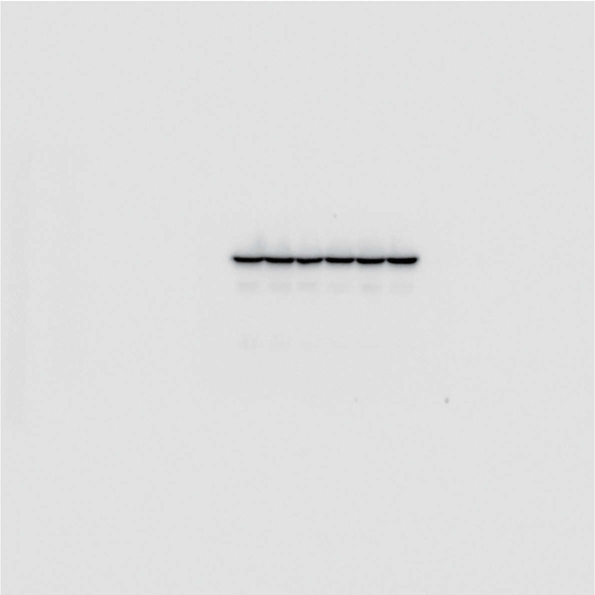

Pgk1

Fig. S2d

Original PVDF membrane

ECL signal-detected blot

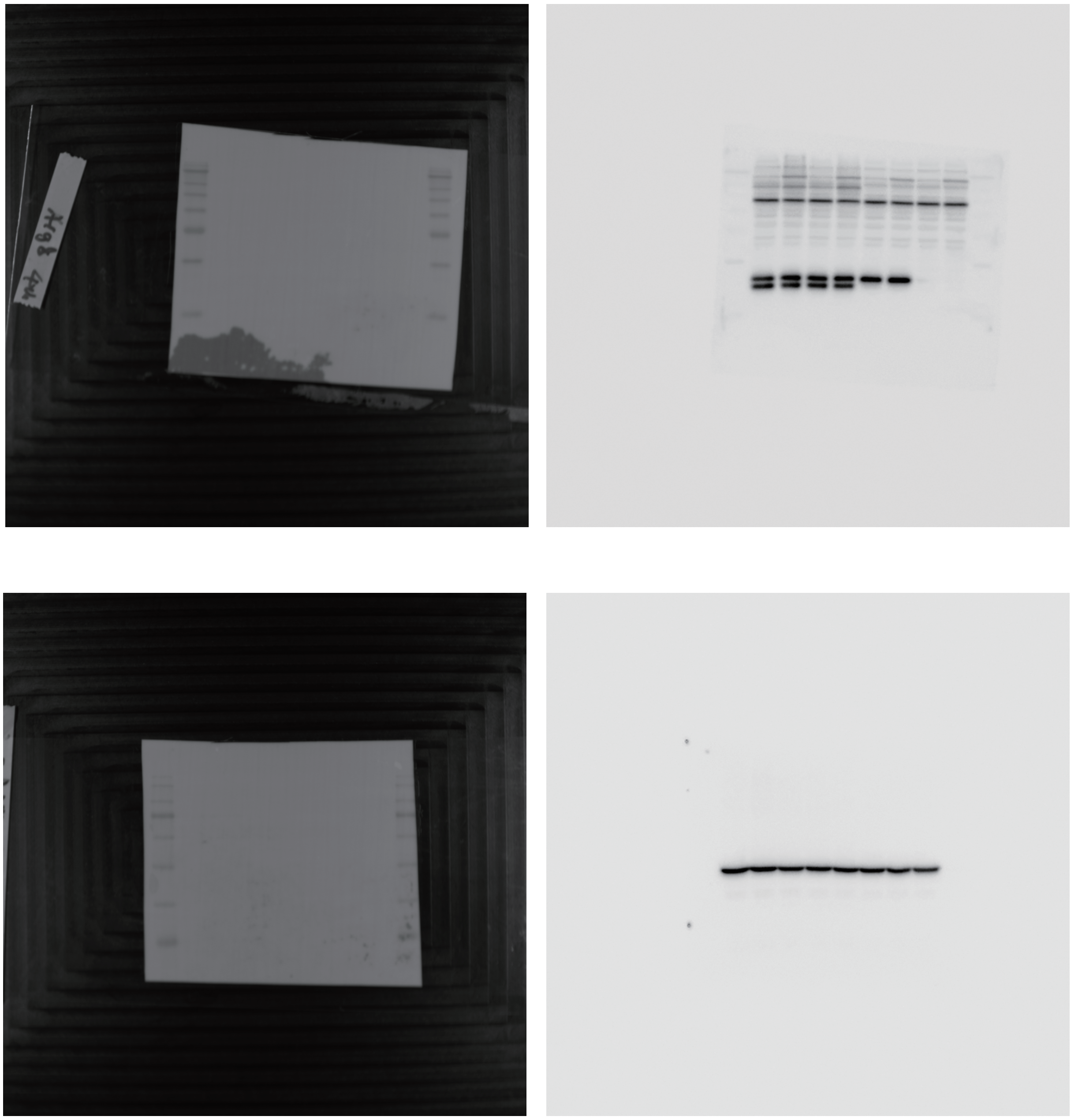

Atg8

Pgk1

Tian and Okamoto
Source data #15

Fig. S2e

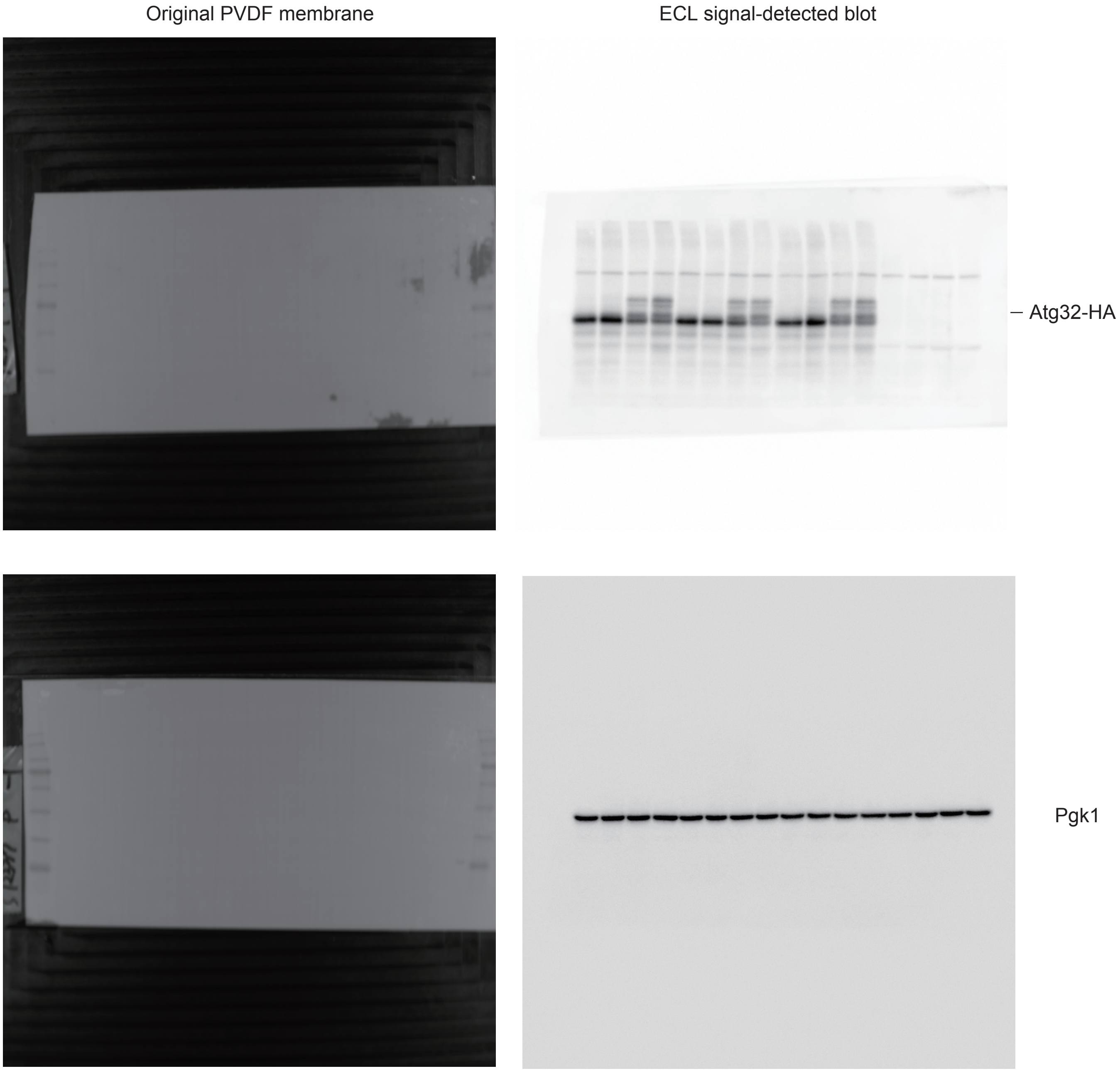

Fig. S3b

Original PVDF membrane

ECL signal-detected blot

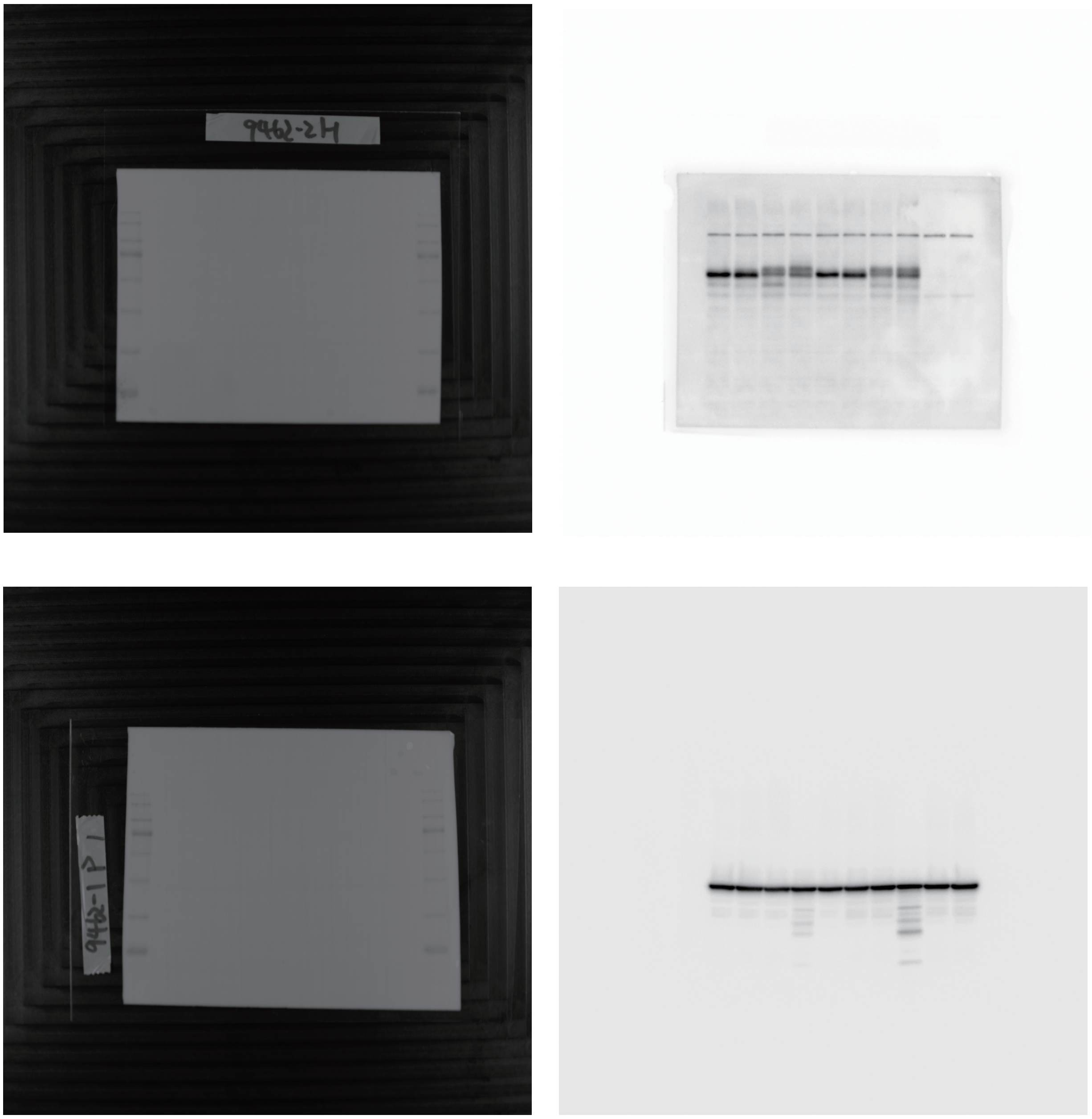

– Atg32-HA

Pgk1

Fig.S3c

Original PVDF membrane

ECL signal-detected blot

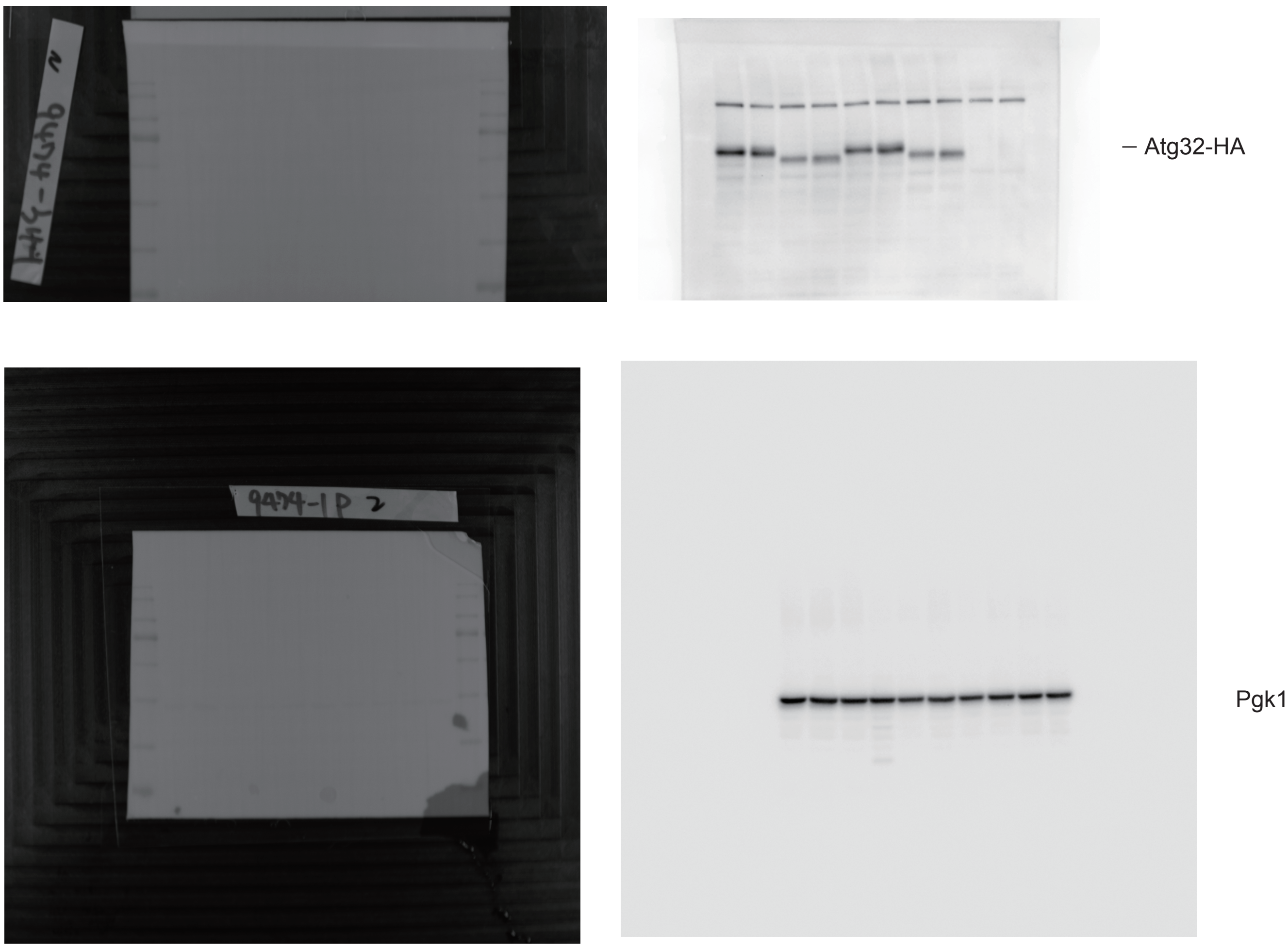

Fig. S3d

Original PVDF membrane | ECL signal-detected blot

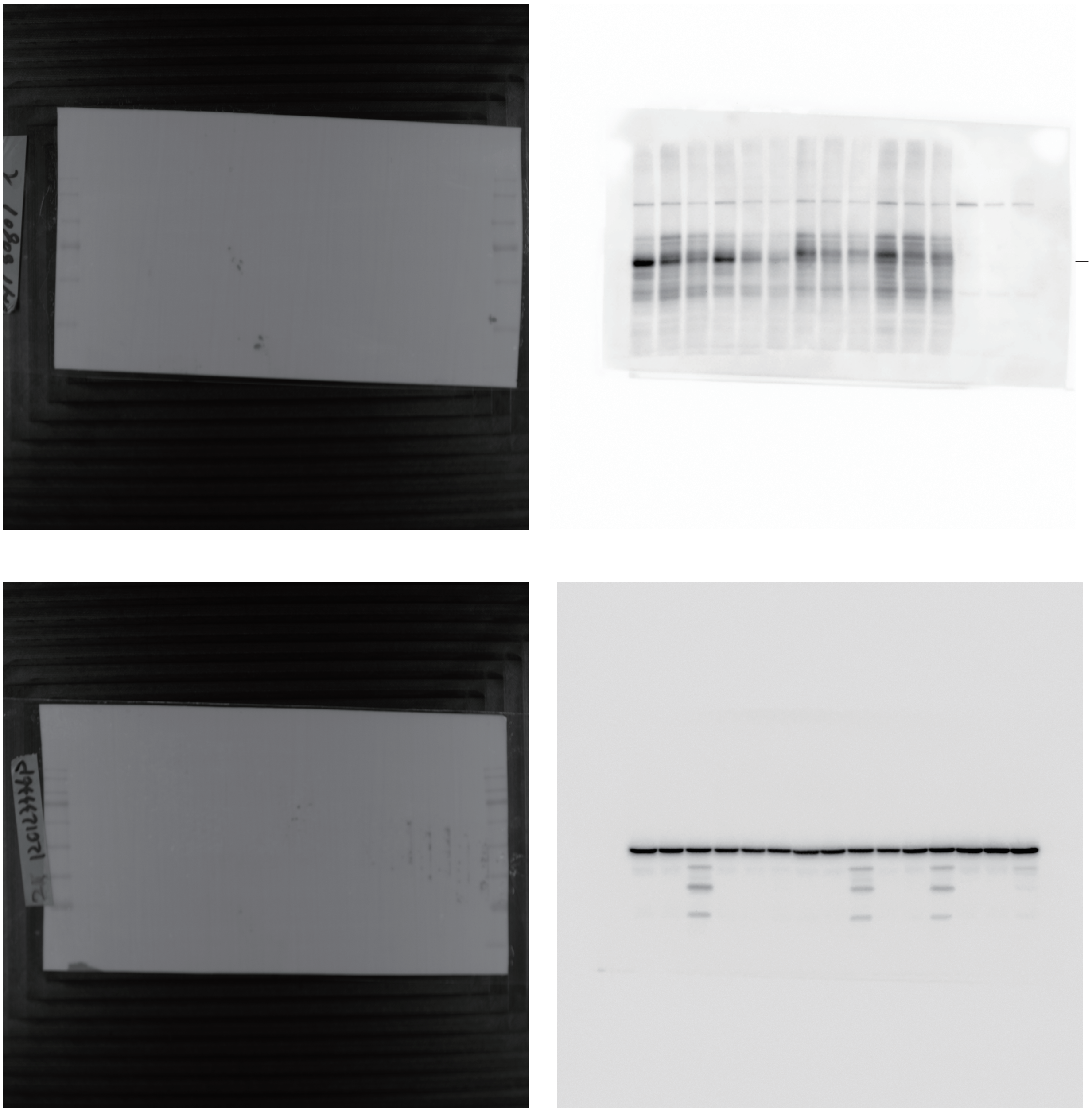

Atg32-HA

Pgk1
